# Receptive fields from single-neuron recording and MRI reveal similar information coding for binocular depth

**DOI:** 10.1073/pnas.2409893122

**Published:** 2025-11-03

**Authors:** Andrew J. Parker, Ivan Alvarez, Alessandro Mancari, I. Betina Ip, Kristine Krug, Holly Bridge

**Affiliations:** ^a^Department of Sensory Physiology, Institute of Biology, Otto von Guericke University, Magdeburg 39120, Germany; ^b^Department Physiology, Anatomy and Genetics, University of Oxford, Oxford OX1 3PT, United Kingdom; ^c^Leibniz-Institute for Neurobiology, Magdeburg 39120, Germany; ^d^Oxford Centre for Functional Magnetic Resonance Imaging of the Brain, Centre for Integrative Neuroimaging, University of Oxford, Oxford OX3 9DU, United Kingdom; ^e^Nuffield Department of Clinical Neurosciences, University of Oxford, Oxford OX3 9DU, United Kingdom; ^f^Department of Translational Research on New Technologies in Medicine and Surgery, University of Pisa, Pisa 56124, Italy

**Keywords:** population receptive fields, binocular vision, magnetic resonance imaging, electrophysiology

## Abstract

The population receptive field (pRF) approach to functional measurement of the sensory properties of magnetic resonance (MR)-identified locations in the human brain was extended to include the third dimension of binocular depth. In total, pRFs were extracted from nine different visual areas (V1, V2, V3, V3AB, V4, V5, V7, Ventral Occipital Cortex: VOC, Lateral Occipital Cortex: LOC) of the human cortex and, where possible, comparisons were made with electrophysiological recordings from homologous areas in the macaque cortex. Human and macaque V1 showed strikingly similar information profiles for the encoding of binocular depth. Further, both human and macaque V5 showed consistent changes in preferred binocular depth of the stimulus, dependent on whether the stimuli were binocularly correlated or anticorrelated. Across the nine areas of the visual cortex explored, the population profiles of pRFs for binocular depth showed evidence of a greater responsiveness to relative depth in higher visual cortical areas, again consistent with the findings from macaque electrophysiology. Overall, the pRF measures of cortical response were more sensitive to fine-scale differences of binocular depth, compared with many existing electrophysiological measures of tuning for binocular depth. Our results show that the pRF method can be extended beyond the characterization of RFs in retinotopic coordinates to reveal higher-order, derived visual properties. The parallels between noninvasive, MR-based measures of pRFs in humans and the electrophysiological recordings of single neurons in experimental animals make a further step toward validation of the pRF methodology.

## Significance

The concept of a receptive field (RF) of a neuron is fundamental in sensory neuroscience, defined by the ordered set of sensory stimuli that reliably induce activation of a neuron. Recent advances in magnetic resonance (MR) imaging have developed methods for isolating cortical activations into RF-like entities. Using binocular vision, we extend the mapping of these MR-based RFs into the third dimension of stereoscopic depth and demonstrate substantial alignments of the resulting response profiles of MR-based RFs with earlier electrophysiological measures of neuronal RFs. The comparison between noninvasive measurements in the human cortex and single-neuron recordings in macaque is an essential step toward validation of the newer methods.

The concept of a receptive field was first proposed by Sherrington (1) to describe the limited area of the skin surface from which the spinal scratch reflex could be evoked. This term has become fundamental in understanding the processing of sensory stimuli by the nervous system. For vision, the classic work of Barlow (2), Kuffler (3), and Hubel and Wiesel (4) revealed that visual stimuli excite action potentials from single neurons of the retina or visual cortex, provided that these stimuli are presented in specific locations in the visual field. Often a specific brightness or stimulus geometry is required, most particularly in the visual cortex.

Exploration of the human visual cortex with functional MRI (fMRI) has been centrally concerned with specifying the spatial organization of cortical responses. Chief among these has been the use of the Blood Oxygen Level Dependent (BOLD) response to identify retinotopic maps (5). Such maps indicate the relationship between an anatomical location in the visual cortex and a horizontal and vertical position in the visual field. Early studies identified that the BOLD response measured at a single anatomical location also depends on the size of the visual stimulus. This suggested that it is possible to measure the spatial dimensions of the population receptive field (pRF) of an anatomical location, as well as the pRF’s corresponding position in the visual field (6).

This approach was formalized by Dumoulin and Wandell (7), who proposed that the cortical response can be described by the combination of a spatial receptive field and a temporal low-pass filter describing the time-course of the BOLD response. The temporal filter is often referred to as the hemodynamic response function (HRF). The HRF parameters can be quantified independently of any measurements of the spatial receptive field and for individual human participants, if so desired. The spatial component is assumed to reflect the pooled activity of neurons at a location on the cortical sheet and is therefore often referred to as a pRF.

Subsequent studies (8–12) have adopted this framework to measure properties of the two-dimensional (2-D) pRF, specified by 3 parameters, namely *x, y* for location and a single parameter to measure RF size in both *x* and *y* directions. These measures reveal encouraging signs of lawful behavior: for example, receptive field size increases with increasing eccentricity in the visual field and this relationship is different in different visual cortical areas. In the primary visual cortex V1, the measured sizes of the 2-D receptive fields for binocular stimulation are consistently larger than those for monocular stimulation, in accordance with the predictions of the binocular energy model for single neurons (9, 12). Other parameters such as motion and color (13) and binocular depth (14) have been tested but, as yet, the results do not always provide a clear interpretation in terms of receptive field properties. The underlying presumption is that BOLD pRF measurements can be related to direct electrophysiological measurements of the visual properties of neurons, available in the form of single- and multiunit activity (SUA and MUA) and local field potential (LFP) in recordings from experimental animals (15–17).

Here, we take a further step toward aligning electrophysiology and MR-based measurements of receptive fields, specifically comparing the encoding of binocular depth by the two measures. This extends modeling of the MR-based pRF beyond the 2-D receptive field to a full three-dimensional (3-D) specification. We isolated the electrophysiological and MR-based responses specific to binocular stereoscopic depth in a controlled way by using random-dot stereograms. We find that the stereo pRFs for the primary visual cortex (V1) deliver maximal information encoding of binocular depth at the fixation plane, in accordance with results from macaque neurophysiology (18). We also show that changes in binocular correlation (19, 20) induce similar changes in both electrophysiological and MR-based responses of pRFs. For cortical area V1, the depth ranges covered by stereo RFs from electrophysiology and MR measurements are in reasonable agreement. However, for many extrastriate areas, the human MR measurements appear to be concentrated within narrower ranges of binocular depth processing. We develop a model for processing of binocular information in a hierarchy of stages that correctly predicts the transformation of stereoscopic information from the processing of absolute disparity in the primary visual cortex to emerging sensitivity to relative disparity in extrastriate cortical areas (21). We conclude that human pRFs for stereoscopic depth show patterns of tuning that differ across visual cortical areas, showing several of the specializations previously identified from electrophysiological recordings in the macaque visual cortex.

## Results

**Fig. 1.**
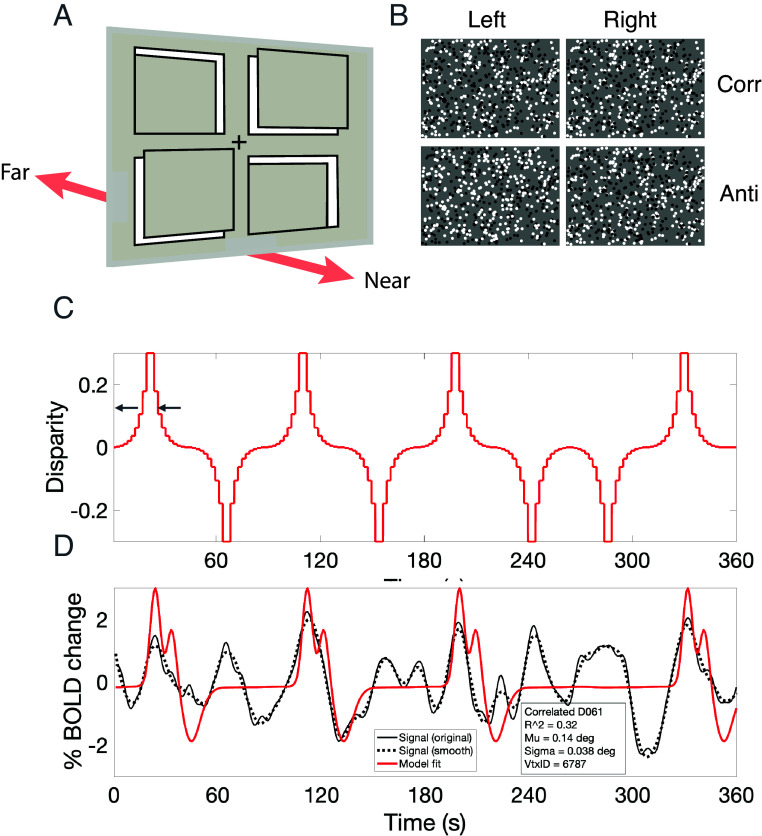
Stimulus configuration and data analysis procedure. (*A*) 3D schematic of stimulus with four quadrants whose binocular depth is varied continuously. (*B*) Examples of random-dot stereo stimuli as displayed in each quadrant of panel *A*; upper pair, binocularly correlated; lower pair, binocularly anticorrelated. In the binocular anticorrelated stimulus, the dots are contrast inverted between the two eyes. Such stimuli can elicit inverted tuned responses, for instance in V1 (19), without a depth percept. (*C*) Waveform of disparity changes over time applied to a single quadrant, the waveform was periodic but occasionally flipped its phase by 180° to guard against adaptation or habituation. The small arrows indicate the preferred disparity of the temporal BOLD waveform below. (*D*) Temporal waveform of the BOLD response; black trace, recorded BOLD response; red trace, fitted model based on kernel of measured HRF. Vertex from left hemisphere of subject D030: fitted model gives preferred disparity (Mu) +0.14 degrees and tuning width (sigma) 0.038 degrees, with R^2^ = 0.32. The model predicts the stimulus changes correctly. Note the double peak in the modeled HRF, corresponding to the double passage of the stimulus, as disparity passes through a value +0.14 degrees and then reverses back through this value shortly after. Image credit: Adapted from ref. 22.

Subjects in the MR scanner viewed a binocular display (Fig. 1) in which the depth of random dots varied systematically in four quadrants arranged around a central fixation point. A contrast detection task was performed to ensure consistent allocation of attention to the display. As binocular depth in the quadrants changed over a range from –0.3 to +0.3 degrees, the cortical BOLD response tracked these changes. BOLD signals were modeled using a one-dimensional pRF model, essentially performing a reverse correlation analysis between the current BOLD response and the value of the stimulus disparity in the relevant quadrant. The temporal relationship between the BOLD measurement and the stimulus was offset in time according to the individual’s HRF. Within a single quadrant, the stimulus disparity varied according to the periodic waveform in Fig. 1*C*, which resulted in a BOLD waveform, Fig. 1*D*, at each cortical point that was excited by the stimulus. The stimulus waveform flipped its phase by 180 degrees from time to time to avoid habituation to a regular sequence of stimulation.

**Fig. 2.**
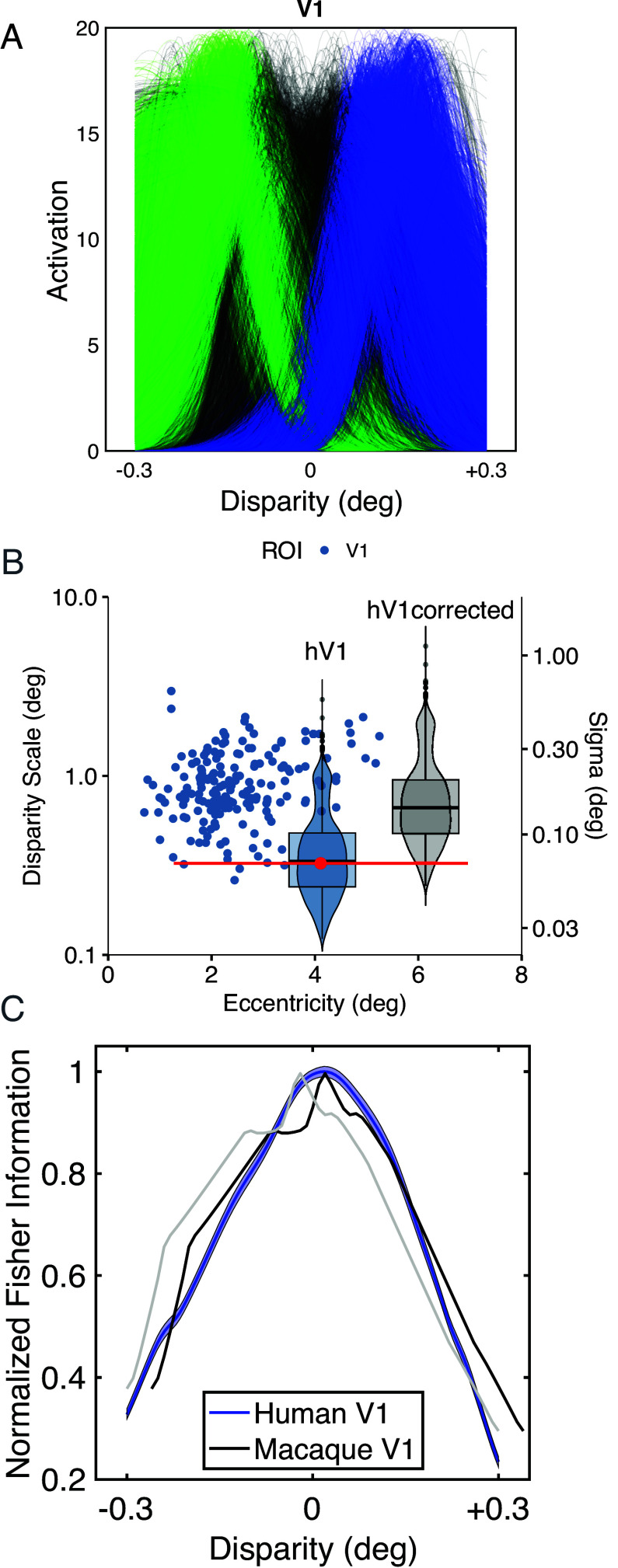
Distribution of pRFs for human cortical area V1. (*A*) Classification of the pRFs into different groups using density clustering. (*B*) Compares tuning widths, disparity scale (21), and sigma (left and right ordinates) against eccentricity. Macaque V1 neurons (blue circles) and human V1 pRFs (violin plot, “hV1” blue, red dot geometric mean of pRF size, located at center of stimulus quadrant, Fig. 1*A*; the red line shows range of eccentricities in the stimulus quadrant). Violin plot (“hV1corrected,” gray), vertically shifted by 2× to compensate for difference in interocular separation of macaque and human and offset by 2 degrees for clarity. Image credit: Adapted from ref. 21. (*C*) Compares smoothed, scaled Fisher information from human V1 pRFs and macaque V1 neurons (replotted from figures 12 and 13 in ref. 18). Blue shading shows 95% confidence limits by bootstrap. Macaque data (black) realigned to peak of human data to remove fixation disparity (original data, gray).

The procedure for pRF identification produces tuning curves for binocular depth at the vertex points of a triangulated surface map of the anatomy of the visual cortex. Retinotopic mapping is used to assign vertices to functional cortical areas. For visual cortical area V1, the outcome is shown in Fig. 2*A*. The responses of 8,463 pRFs pooled across 9 subjects are plotted as ultra-thin lines, to allow any patterning to be evident based on density variations (*SI Appendix*, Fig. S1.6). These V1 pRF tuning curves appear to be concentrated into two groups. This is tested more formally by subjecting the data to a density-based clustering analysis (23). The different clusters are highlighted with blue and green, while unclassified pRF tuning curves are shown in the original gray. In terms of earlier neurophysiological recordings from macaque (24), the blue cluster would correspond to G. Poggio’s “near” and “tuned-near” types of disparity tuning and the green cluster to “far” and “tuned-far” types, although more recent quantification suggests a continuous variation from one neurophysiological type to another (18).

This algorithm assigns data curves to different clusters if there is a deviation from a random distribution. The algorithm requires setting of two parameters: the dimensionless epsilon parameter, which defines a search radius, was set to 25 and the density parameter, which defines the minimum number of points that are required to define a cluster, was set at 7% of the number of available tuning curves. Of the 8,463 valid pRFs recovered from V1, 4,020 were assigned to group G1 (green) and 3,162 to group G2 (blue), with 1,281 remaining unclassified.

To seek validation of this statistical assignment of clusters, the pRF tuning curves were also subjected to a principal components analysis (PCA). The first two principal components (PC1 and PC2) capture greater than 70% of the variability between the tuning curves and the third and fourth PC capture another 21%. *SI Appendix,* Fig. S1.6*C* presents a scatter plot of PC1 against PC2, in which the points are highlighted with colors corresponding to those from the clustering analysis. The assignments from the density-based clustering correspond to differences in tuning shape and specificity summarized by PC1 and PC2.

Further validation of the classifications asserted by the density-based clustering algorithm was conducted with a one-way multivariate ANOVA (MANOVA). Each pRF tuning curve has four parameters, which specify the amplitude, peak location, and width of the fitted Gaussian, together with a measure of the quality of fit. The association between these four parameters and the statistical classification into G1 and G2 was highly significant [F = 6,221, df (8, 16,912), Hotelling-Lawley Trace 5.89, *P* ≪ 0.0001]. The main driver of this association is the difference in the peak positions for groups G1 (green) and G2 (blue). This is obvious by eye in Fig. 2*A* and is confirmed by plots of the average normalized values by group in *SI Appendix*. There is a smaller contribution of width of the fitted Gaussian, essentially reflecting the reality that the peak of the Gaussian is more reliably defined for functions with narrower width. Neither the amplitude nor the performance metric returned from the pRF fits contribute significantly.

### Comparing Depth Ranges Sampled by RFs from Electrophysiology and MR Measures.

The present MR data (Fig. 2*B*) indicates pRFs that are more narrowly tuned than single-neuron data (22), even though the data exhibit the expected broadening of tuning width for disparities away from zero (25, 26). However, in comparing tuning widths of pRFs and neuronal recordings, it is important to use comparable metrics and to consider data matched for visual eccentricity (angular distance from the fovea).

Unlike the Gaussian fits to pRFs used here, previous neurophysiology used a Gabor function, the product of a sinusoidal function with a Gaussian, to describe cortical tuning curves. Tuning widths were summarized by the frequency of the fitted Gabor [“disparity frequency” (18, 27)] or its reciprocal [“disparity scale” (21)]. In reality, disparity tuning curves have weak side-lobes when described by Gabor functions: in macaque V1, the mean number of sinusoidal cycles in the tuning curve is 0.25 (see figure 8 in ref. 28). Consequently, Gaussian fits are adequate for the majority of macaque V1 (28) and V5/MT (29) neurons. To compare disparity scale and single Gaussian fits, we recently presented an analysis based on a pair of Gaussian curves, one inverted with respect to the other and separated from each other by the full-width at half-height (2.35 σ), so that they approximate the half-period of a sinusoid (22). This results in a conversion factor of twice this value 4.7, to align earlier neurophysiological measures reported using disparity scale with the current MR-based pRF measures based on Gaussian fits.

Both measures are employed to summarize the outcome graphically in Fig. 2*B*, with points for each neuronal measurement in V1 recordings, adapted from ref. 21. The human pRF results from V1 from this study are shown by the superimposed “violin” plot. This is centered at the red dot, which shows the visual eccentricity matching the centers of the quadrants in the mapping stimulus (as in Fig. 1*A*), with the horizontal red line showing the range of eccentricities in the stimulus. The vertical distribution of the violin plot (“hV1”) summarizes the spread of human V1 pRF widths, in terms of both disparity scale (left y-scale) and sigma (right y-scale). There is substantial overlap between the electrophysiology and MR-based measurements, but the alignment is not perfect.

The distribution of pRF widths from human MR measurements appears shifted toward narrower tuning compared with macaque neurophysiology. This may reflect a greater sensitivity of human stereo system, compared with macaque, on account of the greater eye size and interocular separation in humans (22). The gray violin plot (“hV1corrected,” offset by 2 degrees for clarity) is shifted vertically by a factor of 2 to reflect the differences between the species. This brings the disparity scale of MR-based pRFs and macaque electrophysiology into alignment for V1 responses, although it would be possible to argue for scaling factors other than 2. Ideally, this comparison would require direct measurements of the pRF in macaques with simultaneous recordings of macaque neurophysiology.

#### V1 pRFs support high sensitivity to binocular depth at the fixation plane.

Theoretical and experimental studies have shown that sensory neurons deliver the most precise information about stimuli when small changes in the stimulus evoke reliable changes in neural activation (30–32). This insight has been expanded with the development of a framework based on Fisher information for evaluating encoding in sensory populations. Fisher information summarizes the accuracy with which the value of the sensory stimulus is specified by the conjoint activity of a population of neurons (33, 34). Its full calculation requires knowledge of the variance and covariance of neuronal activity on time scales that are relevant to perceptual decisions. Encoding of binocular depth follows these general principles (27, 35, 36).

To apply this framework to the pRFs obtained here, it is necessary to make some assumptions. A potential limitation is that there are no empirical measures of variance or covariance available. However, each contributing pRF was required to pass a statistical threshold for reliability during the data preparation, so some degree of homogeneity has been enforced. Our assumption here is that the variance is the same for each contributing pRF, and in the analysis, we set this value to 1. In respect of covariance, it can be noted that the vertices for the pRFs are spaced sufficiently far apart on the cortical surface that shared noise is likely to be low. Moreover, the noise correlation structures that adversely affect perceptual discriminations about binocular depth occur on much shorter time scales than the integration time of the hemodynamic response that generates the pRF data (36).

The contribution of a single pRF tuning curve to Fisher information is calculated by taking the change in the pRF response evoked by a small change in binocular disparity, which is mathematically the differential of the pRF tuning curve. Responses from multiple pRF tuning curves are combined by summing the absolute values of the differential at each stimulus disparity along the tuning curves and smoothing the resulting average. Thus, both positive and negative slopes contribute Fisher information that supports sensory discrimination.

The resulting distribution of Fisher information as a function of binocular disparity is shown in Fig. 2*C* for human V1 (hV1). The distribution peaks at zero disparity, indicating that the set of recorded pRF tuning curves delivers the highest information about disparity around the zero value. The neural population has the greatest density of steep slopes, around the fixation plane, both in terms of the slope of individual pRF tuning curves and the numbers of identifiable pRFs. For Gaussian tuning curves, this outcome is delivered by a distribution of individual tuning curves that have peaks at nonzero disparities. This distribution provides for maximum sensory information about binocular depths near to zero disparity, close to the fixation plane.

Fig. 2*C* compares Fisher information from macaque single-neuron data [see figures 12 and 13 in Prince et al. (18)] and human pRF data. The two curves have been normalized to a peak of 1 and a small offset of ~0.03 degrees between the peaks was eliminated, as this likely reflects a slight difference in fixation disparity between the two datasets (compare gray and black plots in Fig. 2*C*). There is a tight correspondence between these two different measures from the primary visual cortex (V1) in the two different primate species. The similarity of the two plots in Fig. 2*C* provides validation of the MR-based pRF methodology.

#### Responses beyond the primary visual cortex.

**Fig. 3.**
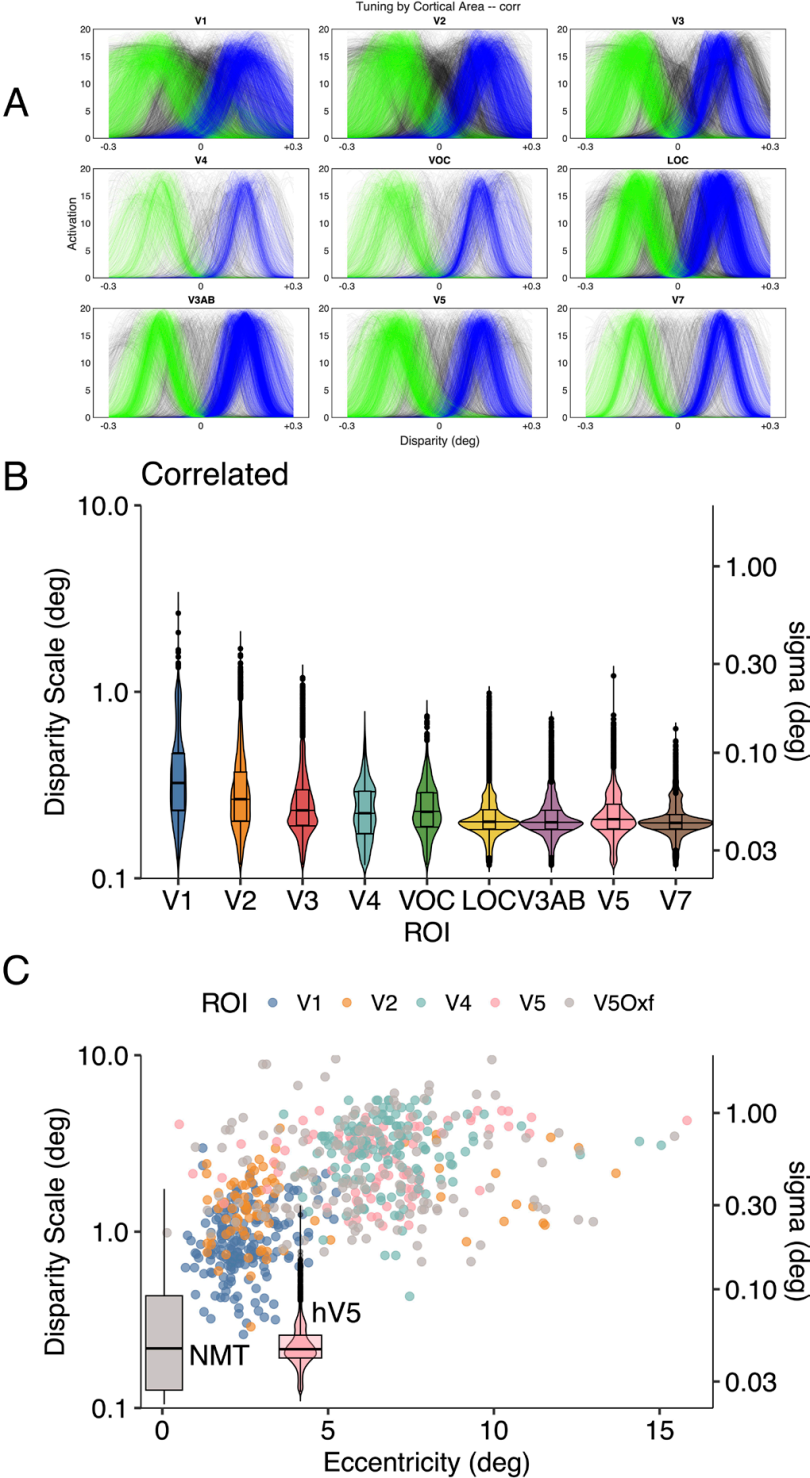
(*A*) pRF measures of human cortical responses to correlated disparity from all 9 identified visual areas after applying density-based clustering. (*B*) Violin/box plots of distribution of tuning widths for correlated disparities by cortical area, showing both disparity scale and sigma. (*C*) Tuning widths of macaque single neurons from areas V1, V2, V4, and V5 from ref. 21 plus additional V5 data (V5Oxf) from ref. 37 with superimposed violin plot of hV5 pRFs located at center of stimulus quadrant and boxplot distribution of NMTs for disparity discrimination by single neurons from macaque V5 (data reproduced from ref. 38, figure 6, location on the x-axis at arbitrary point). Anticorrelated results in *SI Appendix*. Image credit: Panel *C* adapted from ref. 21.

In total, retinotopic mapping identified 9 visual cortical areas and the pRF analysis procedure was also applied to measurements from all these other visual cortical areas. The results are summarized in Fig. 3*A*, which shows pRF tuning curves for binocularly correlated disparity, colored according to the classification with the density-based clustering algorithm. The most striking aspect of these data is that the separation of pRFs into distinct groups is present in all visual areas but is more pronounced in higher-order visual areas, resulting in more bimodal population responses in these areas.

A second feature of the pRF tuning curves is summarized in Fig. 3*B* by the violin plots of disparity scale for all 9 identified cortical areas. Measured disparity scale becomes smaller in higher-order cortical areas, in comparison with V1. The degree of narrowing is quite subtle, typically greater than a factor of $\sqrt{2}$ but never more than 2. However, this trend contrasts with the reported outcome from monkey neurophysiology, which indicates that disparity scale increases between V1, V2, V4, and V5/MT (21).

Fig. 3*C* shows a previously published summary of electrophysiological measures of disparity scale (21) plus some extra measurements for V5/MT from Oxford recordings (V5Oxf) (20). A violin plot of the MR-based pRF measurements from human V5 (hV5) is superimposed for comparison. The discrepancy is obvious. The pRF mapping procedure is revealing disparity interactions on a much finer scale than the tuning curve, but there are some methodological differences. The chiefs of these are the range of disparities tested and variety of stimulus configurations.

The neurophysiological studies that have provided estimates of disparity scale have typically measured the tuning functions of neurons with single-dot planes. The disparity often ranged over a degree or more of visual angle, sometimes with a sampling interval between tested disparities of 0.4 degree, which is larger than the total range tested here (29). Fewer recordings are available from V5 neurons using small changes of disparity within the vicinity of the fixation plane. In one case, the test stimulus was pairs of moving planes of dots slightly separated in depth by binocular disparity, forming the front and back surfaces of a rotating cylinder (20, 38–40). Thresholds for discrimination of binocular depth by these single isolated V5 neurons are overlaid in Fig. 3*C* as a box-plot, which uses the neurometric threshold (NMT) values from figure 6 in ref. 38. These NMT values overlap the human pRF sizes found here, very different from the standard electrophysiological measures of disparity scale. While there are still unresolved questions about which stimulus configurations deliver this fine-grain discrimination, we conclude that when the sensitivity of V5 RFs is probed with configurations displaying small relative disparities, there may be up to a 10-fold improvement in estimated performance.

##### Binocularly anticorrelated visual stimuli.

The procedure for estimating the cortical pRFs during viewing of binocularly anticorrelated stimuli followed closely the procedure for correlated stimuli. The same spatial distributions of dots and periodic disparity waveform were displayed to the participants. The same contrast detection task was required to ensure consistent attention to the stimuli, and the same statistical processing pipeline was used for analyzing the outcome of the MR measurements. The individual pRFs for binocular anticorrelation are shown by cortical area in *SI Appendix*, Fig. S3.1*A*. Tuning for depth persists in early cortical visual areas, as found previously in electrophysiology but the response to binocular anticorrelation is weaker and less clearly structured, notably in higher-order visual areas, again consistent with electrophysiology (41, 42). A spatial map of the distribution of significant pRFs due to anticorrelated activity is found in ref. 22. Plots of the disparity scale of the fitted pRFs for anticorrelated stimuli are also shown in *SI Appendix*, Fig. S3.1*B*. In comparison with the results for correlated stimuli (Fig. 3*B*), there are no obvious trends or differences across cortical areas.

##### Comparing human and macaque cortical area V5.

A more detailed insight into the relationship of human MR-based pRFs with neuronal physiology is available from V5, as there are extensive sets of electrophysiological recordings from macaque V5 to correlated and anticorrelated random dot stereograms (20). First, for binocularly correlated stimulation, we find that both MR-based pRFs and electrophysiological recordings have an overrepresentation of near disparities, in front of the fixation plane. This bias has been observed previously in macaque recordings from V5 [see figure 5 in Parker et al. (43) and figure 8 in DeAngelis and Uka (44)]. In both cases, many of these neurons have spatial receptive fields in the lower visual field. The same bias is also found from a meta-analysis of multiple recording studies from macaque V1 (45), but is smaller in size.

**Fig. 4.**
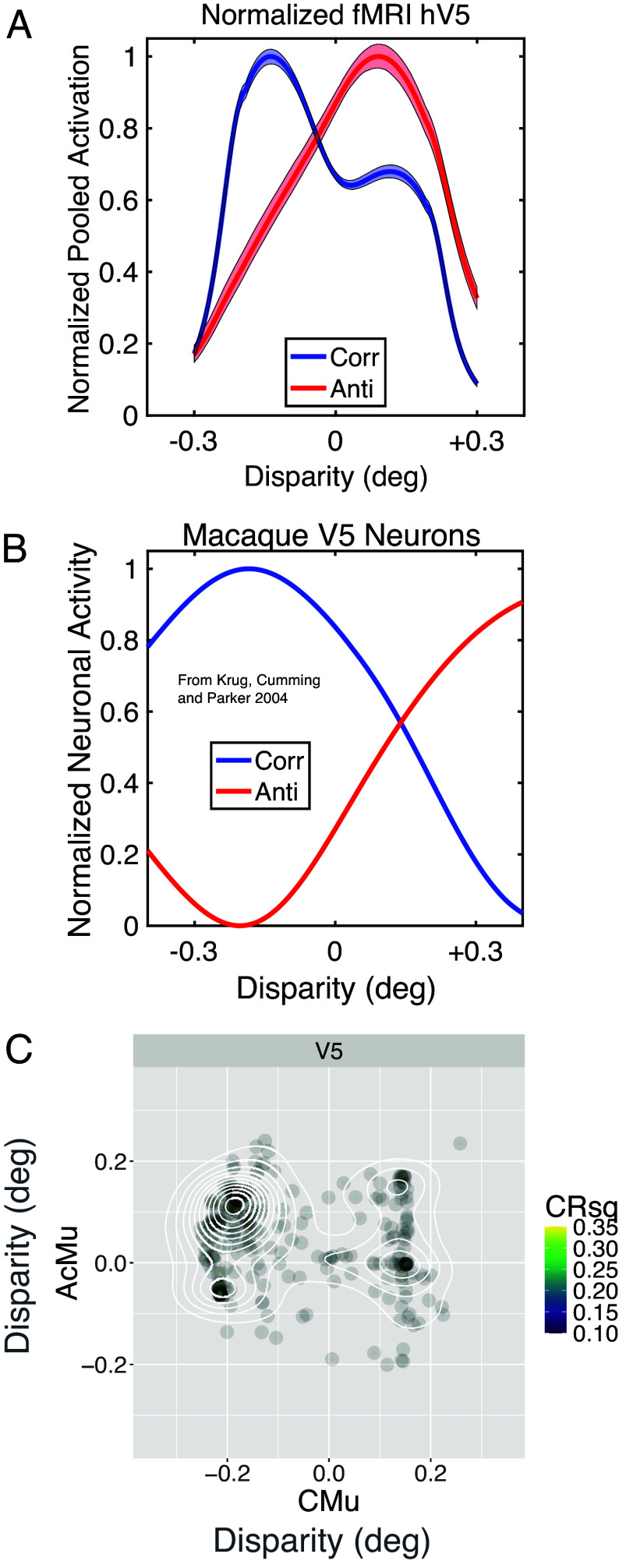
Neural population responses of V5/MT to correlated and anticorrelated stereograms. (*A*) MR-based pRF data from the human cortex in this study. Each curve normalized within range 0 to 1. Shaded regions show 95% confidence limits from bootstrap. (*B*) Neuronal data from macaque V5, previously reported in ref. 20. (*C*) Vertex-by-vertex (N = 1,440) comparison of preferred disparity for correlated (CMu) and anticorrelated (ACMu) stimulation, showing pRFs with R^2^ > 0.1. Binomial test of opposite-sign (*Upper* *Left* and *Lower* *Right*) vs. same-sign preference shows imbalance, mean probability 0.645 (0.620 to 0.670, 95% confidence limits, *P*-value < 2.2e-16).

The blue curves in Fig. 4 are the population responses to binocularly correlated stimuli. These are formed by averaging the extracted tuning curves for each disparity within the tested range, with the electrophysiological responses weighted with a square root transform as in figure 12 of Prince et al. (18). Both human and macaque curves peak at a negative disparity of about –0.2 degrees, corresponding to a population preference for near depths. By itself, this bias could be just an interesting coincidence, but the presence of the bias provides a simple prediction for the population response to anticorrelated stimuli. The binocular energy model (46) predicts that the bias toward near disparities observed for correlated stimuli should emerge as a bias toward far disparities with anticorrelated stimuli (19, 47).

To set aside differences in response amplitude in comparing macaque neuronal data with human pRFs, the normalized responses are compared in Fig. 4. The change from correlation (blue) to anticorrelation (red) yields the predicted change from near to far in the peak of the population responses for both human MR-based pRFs and macaque electrophysiological data (19, 20). A more detailed picture for the MR data emerges from a vertex-by-vertex comparison of the disparity preferences of the pRFs. Fig. 4*C* shows a cluster of points in the upper left quadrant, corresponding to pRFs that prefer near disparities for correlated RDS patterns and far disparities for anticorrelated stimuli. This shift of peak preference is predicted by the binocular energy model (19) and is a further significant parallel between MR-based pRFs and electrophysiology.

Comparison of the amplitudes of the anticorrelated and correlated responses for pRFs is less straightforward. Modeling shows nonlinearities may contribute in different ways, so that reductions in amplitude and shifts in the peak position of the anticorrelated response are both possible (47). Earlier electrophysiological results summarized the differences between correlated and anticorrelated responses with an amplitude ratio, which can be averaged across multiple recordings. Calculating the amplitude ratio for the population of human V5 pRFs suggests that the response to anticorrelated stimuli for fMRI data is lower than that for electrophysiological recordings (0.258 for human pRFs and 0.50 for macaque V5). Interestingly, the calculated difference for psychophysical filters in humans [amplitude ratio of 0.16 and 0.2 for two subjects; see Neri et al. (48)] is about the same as that for human pRFs for V5.

For the other 8 visual areas, the bias toward overrepresentation of near disparities is less evident or even absent. This means that the population profile for these areas does not offer such a straightforward prediction for comparison of responses to correlated and anticorrelated stimuli. For V1 specifically, the neurophysiology shows a fraction of neurons whose firing is suppressed for correlated RDS patterns at disparities near to the fixation plane, so-called “tuned inhibitory” neurons (49). These neurons are excited by the corresponding anticorrelated stimuli. Further experimental measurements, using binocularly uncorrelated dot patterns as a baseline stimulus, will be needed to pinpoint the exact size of the response to anticorrelation. Current data for all areas are summarized in *SI Appendix*, Fig. S4.1.

### Transformation of the Cortical Response to Disparity from V1 to Higher Areas.

Binocular depth is often presented as the difference in location of a single external feature in the left and right eyes that arises geometrically due to the horizontal separation of the eyes. This is correct for V1 (50) but has limitations in that retinal locations change when the eyes converge or diverge. The perceptual response of macaques and humans to binocular depth is dominated by relative disparity, which is the difference in binocular depth between two visible features. Westheimer (51) established that depth judgments with simple stereoscopic stimuli are some 10 times more sensitive when relative disparities are present than when they are eliminated. Single-neuron physiology in macaques has found that encoding of relative disparities is not found in primary visual cortex V1 (50), but emerges in the secondary visual area V2 (52) and beyond (37, 53). The presumption from these neuronal studies is that higher-order processing acts upon the outputs of V1 to deliver sensitivity to relative depth (21).

Here we integrate computational models of how the visual cortex responds to relative disparity with a mechanistic interpretation of the MR-based pRF measures. Earlier work in macaques has made simultaneous measures of single-neuron activity, the LFP and BOLD activation in the visual cortex (15). The LFP is thought to reflect the summed electrical activity of many synaptic potentials. During simultaneous recording, the relationship between BOLD and LFP was tighter than the relationships between neuronal spiking activity and either BOLD or LFP (17). A separate line of reasoning has concluded that the relationship between synaptic activations of single neurons and their spiking activity is described by a threshold below which no spikes are generated followed by squaring output nonlinearity. This relationship is sometimes described as half-squaring (54). For binocular activations, this relationship has been computationally estimated from neuronal recordings in cats as having an exponent α of 2.0 to 2.4 (55).

**Fig. 5.**
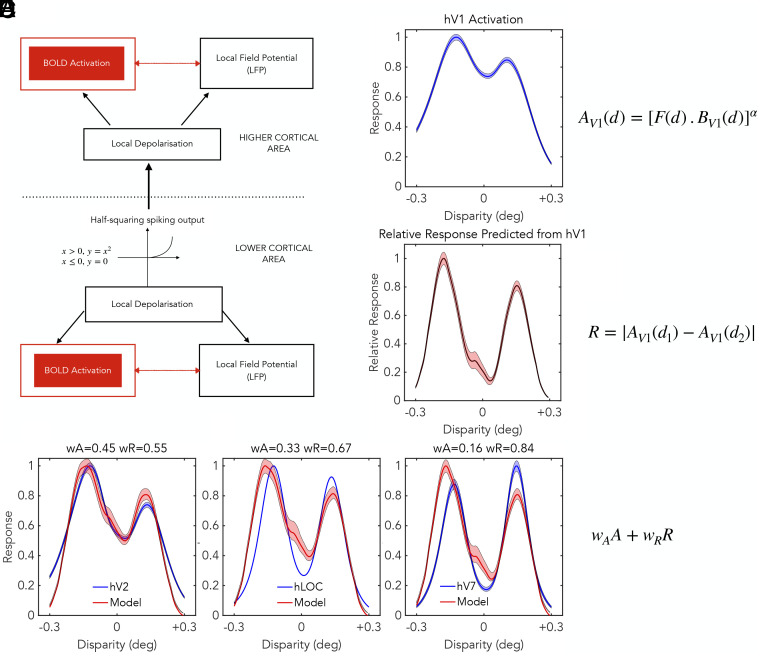
Modeling framework for building responses to relative disparity by the transformation of signals from one cortical area to a higher cortical area. Within lower and higher cortical areas, the BOLD response is related to the local depolarization, as is the LFP. The signal from lower area to higher is transmitted with spiking output and therefore passes through a half-squaring relationship. Output of V1 as a function of disparity *A*_*V*1_(*d*) is calculated from BOLD response $B_{V1}$ weighted by Fisher information F, and raised to power α, here set to 2. Predicted response to relative disparity R is given by the difference of V1 responses, *A*_*V*1_(*d*_1_), *A*_*V*1_(*d*_2_) to two nearby disparities (*d*_1_, *d*_2_). *Lower* three panels show comparison of model against measured activity for 3 cortical areas, hV2, hLOC, and hV7. Model assumes higher cortical areas respond with a weighted sum of responses to absolute and relative disparity $w_{A}A+w_{R}R$. Panels show estimates of weights for each cortical area. Shading shows 95% confidence limits from bootstrapping. The response of higher-order cortical areas is more strongly dominated by relative disparity. See main text for descriptions of panels *A*–*F*.

The block diagram in Fig. 5 summarizes these relationships in schematic form. Incoming synaptic activity is pooled onto groups of target neurons and creates a depolarization of these neurons. These depolarizations lead to spiking activity of the neurons, which is subject to the half-squaring relationship. The same depolarizations occur in multiple target neurons and, after spatial pooling, lead to a signal that is manifest as a change in LFP. When comparing MR-based pRFs with neuronal RFs from a single cortical area, a fundamental difference is that the MR-based pRFs are derived from BOLD and the neuronal RFs are derived from spiking activity. For this reason, the relationship between a MR-derived pRF and neuronal activity is expected to have a half-squaring format.

We developed a simple model (Fig. 5*A*) that takes the measured profile of V1 BOLD activity as its input and predicts the BOLD response of a higher-order area that might contain some neurons sensitive to relative disparity. The model replicates the two stages of processing found from the results of single-neuron recording. The pRFs in V1 (Fig. 5*B*) are assumed to be dominantly sensitive to the absolute depth of single visual features, responding strongly whenever there is simultaneous binocular stimulation of the two retinas (50, 56, 57). The aim is therefore to build sensitivity to relative depth out of these V1 elements (21, 51, 58). V1 is more accurate at representing binocular depths around zero disparity, close to the fixation point, which is reflected in the model by a set of weights set by the Fisher information. The model takes the BOLD signal of V1 pRFs *B*_*V*1_(*d*) as a function of disparity d, weights the outputs according to the Fisher information $F\left(d\right)$, and then passes the weighted outputs through a half-squaring mechanism to simulate the spiking activity of V1 $A_{V1}\left(d\right).$

To form a higher-order neuron sensitive to relative disparity requires the comparison between pairs of these V1 neurons each of which is maximally sensitive to different values of absolute disparity. Thomas et al. (52) used an extension of the binocular energy model (57) to create higher-order neurons that are sensitive to the relative disparity $\psi =d_{1}-d_{2}$. We assume that the higher-order cortical areas contain some neurons sensitive to $d_{1}-d_{2}$ and others sensitive to $d_{2}-d_{1}$, which gives the higher-order population a sensitivity to relative disparity regardless of the sign of the difference. The term R in Fig. 5 is straightforwardly the unsigned difference between activity of two neurons, $A_{V1F}$ and $A_{V1B}$, with each neuron maximally sensitive to different absolute disparities, $d_{F},d_{B}$, where the subscripts F and B refer to two regions of the higher-order receptive field, tentatively labeled foreground and background. In *SI Appendix*, we expand on the relationship between this calculation of R and the energy model (52). At this stage, we therefore have two quantities $A_{V1}$, which are the responses of V1 neurons to the absolute disparity in their receptive fields and R, which is the predicted response of a higher visual area that is exclusively sensitive to relative disparity. See *SI Appendix*, section S2 for additional details.

To capture the full range of possible sensitivities to absolute and relative disparity, the model prediction for higher-order cortical areas is based upon a weighted sum,

$w_{a}*A+w_{r}*R$, where A and R are the absolute and relative disparity predictions respectively. The weights sum to unity, $w_{a}+w_{r}=1$, so a single parameter determines the balance between absolute and relative disparity. A simple minimum search was performed to find a least-squares fit of the weighted prediction to the average population response recorded for extrastriate areas.

There are three notable aspects of the human pRF results in higher-order visual areas. First, the profile of activation in higher-order visual areas generally shows a more distinct separation into two groups that are tuned to nonzero disparity values (Fig. 3*B*). Second, the tuning widths become narrower in higher-order visual areas (Fig. 3*C*). Here, we show that both these changes are compatible with increased sensitivity to relative disparity. Third, while the disparity scales of pRFs recovered from hV1 are closely similar to neuronal RFs from macaque V1, pRFs recovered from higher-order human cortical areas do not exhibit some of the larger disparity scales found with neuronal recordings in macaque. This last point is addressed in *Discussion* section.

Fig. 5 *D*–*F* shows both the experimentally measured profiles of activity and the model predictions for three cortical areas: hV2 and two higher-order areas, hLOC in the ventral stream and hV7 in the dorsal stream. Given the simplicity of the model, it performs well in describing the main features of the response profiles, most evidently for hV2 for which the fit is very good. The model has difficulty with one aspect of hV7. The pooled responses from hV7 show a bias toward far disparities, whereas the model prediction uses the baseline of pooled hV1 responses, which are slightly biased toward near disparities (Fig. 5).

The profile for hV2 is consistent with a degree of specialization for relative disparity, with $w_{r}=0.54$. The profiles of pRF data for hLOC and hV7 are consistent with a greater weighting for relative disparity with respectively $w_{r}=0.67$ and $w_{r}=0.84$, closer to the perceptual response (58, 59). For these two higher-order areas, there is independent evidence linking them to perceptual processing of stereoscopic depth (60–62). The overall trend of pRF responses compared across human cortical areas is similar to that derived from single-neuron responses recorded from the macaque visual cortex: visual areas deeper in the processing sequence show a greater responsiveness to relative disparity rather than absolute disparity (21).

With regard to the narrower tuning widths in higher-order cortical areas, the mathematical structure of the energy model computation predicts that a higher-order tuning should be narrower than the component sources; see Appendix B in ref. 28 and *SI Appendix*, section S2. For Gaussian tuning curves, the degree of narrowing is $1/\sqrt{2}$. Thus, the prediction is if higher-order cortical areas compute relative disparity using a higher-order energy computation, the disparity scale (or equivalently, sigma) should be narrower than that of V1 by this theoretical factor. Fig. 3*B* shows that some cortical areas (specifically V4, VOC) conform to this prediction, whereas others show a greater degree of narrowing. This greater narrowing could be due to disparity processing through multiple hierarchical stages and may be approximated by raising the exponent α to values greater than 2.

## Discussion

We have extended the MR-based method of measuring pRFs in the human visual cortex beyond the measurement of pRF properties in purely retinotopic coordinates. Responses in the human cortex for the third dimension of binocular depth were isolated using a MR-based, pRF analysis of BOLD activity. Like previous MR studies (63), our experimental procedure explored a limited range of disparities close to the binocular fixation point, so that we could be confident that all participants had a continuous percept of stereo depth without any breaks in binocular fusion. This may mean that we have missed some pRF tuning for binocular depths that lie outside these limits. For V1, the comparison of tuning widths for pRFs and macaque physiology indicates that this is limited (Fig. 2*B*) but for other areas this could be greater (29), although differences in visual eccentricity and other stimulus conditions may frustrate direct comparisons.

These human MR-based pRFs show four fundamental similarities with neuronal responses to binocular depth recorded electrophysiologically from macaque monkeys. First, for cortical area V1, the Fisher information about absolute disparity conveyed by the human and macaque neural populations is closely similar (Fig. 2*C*). Second, also for V1, the pRF method yields measures of tuning width (disparity scale) similar to those from macaque neurophysiology (Fig. 2*B*). Third, with binocularly anticorrelated stimuli, the cortical response of the MR-based human pRFs is considerably weaker than that for binocularly correlated stimuli, just like the neurophysiological results from the macaque cortex. A specific insight comes from the population response of human cortical area V5, which shows a bias for near disparities that reverses when the stimulus is changed from correlated to anticorrelated. These two features are also found in the electrophysiological activity of macaque area V5. Fourth, the profile of pRF activity in higher visual cortical areas is consistent with the steady emergence of sensitivity to relative disparity. In the highest cortical areas tested, the weighting of the signal toward relative disparity is similar to the weighting inferred from perceptual experiments (58, 64), whereas in V2 the pRF response to relative disparity is less strong, as found electrophysiologically (52).

### Information Content of V1 Signals About Binocular Depth.

A full calculation of Fisher information requires knowledge of variance of the signals from individual receptive fields, and further the covariances between the signals (33, 36). Insight into the variances is available from the neurophysiology. The best model suggests that spiking activity is related to LFP with an accelerating square-law nonlinearity (54, 55). If this is correct, then to maintain a flat variance from all pRFs, the signals from these pRFs can be combined as if they were signals from neurons with a square root transform applied to the neurons’ firing rates.

For covariances, it is important to consider separately the signal correlations (common variation caused by similar selectivity and responsiveness to the stimulus) from noise correlations (typically assumed to be shared input from noisy source in the neuronal circuity). At the distance separating one vertex from another in the pRF data, the noise correlations are likely to be weak, possibly negligible. However, a limitation of MR-BOLD measurements is that neuronal signals are passed through the slow time-course of the hemodynamic response. Macaque physiology shows that during the viewing of random-dot stereo stimuli interneuronal correlations in areas V1 and V4 are on the time scale of tens of milliseconds and appear to be linked to receptive field overlap (36). Optical imaging suggests longer-range functional interactions in the cortex, but these are largely driven by signal correlations (65, 66).

V1 appears to be well suited to the establishment of local binocular correspondence, rather than binocular depth processing. This is evident in the responses to anticorrelation and the response to absolute disparity. The greater portion of neuronal resources in V1 is devoted to processing binocular information from objects that lie in the depth plane on which both eyes are simultaneously converging (67).

### Disparity Scale across Multiple Visual Cortical Areas.

Macaque neurophysiology shows sensitivity to binocular disparity at scales much larger than revealed in the current MR-based pRF measurements. There are some fundamental differences in the two measurement techniques. Notably, neurophysiology and MR-based measurements have different baselines. For neurophysiology, the baseline is typically the resting firing rate of the neuron, whereas the pRF technique has an effectively floating baseline, since the fitting procedure is arranged to be sensitive to a change in BOLD brought about by a change of disparity. Critically, pRF methods are only effective if something in the neural responses changes when binocular depth is changed. Consider a cortical site with receptive fields that are predominantly near/far in their disparity tuning. At large nonzero binocular depths, the underlying neuronal response is driven by two sets of neurons, one responding to far depths and another responding to near depths. For both types of neuron, there is a large range of binocular depths over which there is not much change in response. For cases such as these, the implementation of the pRF method used here is not likely to reveal much tuning. Other approaches would be needed, such as comparisons of binocular correlation against a baseline response to a binocularly uncorrelated stimulus.

Given that the present pRF data indicate interactions over small disparity scales that become progressively smaller in higher-order visual areas, we have considered carefully whether there are some fundamental methodological flaws that may be biasing the outcome. Poor quality data fitting might be a source of bias. Against this is the finding that anticorrelated responses are generally weaker than correlated responses but do not show the same trend across visual areas. Also, direct inspection of the relationship between peak or width of fitted tuning and quality of fit (R^2) shows no evidence of bias emerging at lower values of R^2 (*SI Appendix*, Fig. S4.2).

A second line of evidence against fundamental methodological problems is that the pRF method produces good quantitative alignment with macaque neurophysiology for disparity scale measurements in cortical area V1. For areas such as V5, which present a discrepancy, there is separate neurophysiological evidence of fine-scale disparity processing (Fig. 3*C*). For V5 specifically, these responses of single neurons to fine-scale disparity have been validated with measures of significant choice probability (40) and positive outcomes employing causal intervention in the form of electrical microstimulation (68, 69). In summary, we conclude that these pRF measures for hV5 present further evidence for a component of V5 responsiveness that is sensitive to fine-scale disparity. Two cautionary points need to be added to this conclusion. First, knowledge about which stimulus configurations promote fine-scale disparity sensitivity in V5 neurons is incomplete. Second, the correspondence between macaque V5 and human V5 is not thought to be exact, with components of hV5 responses that are certainly variable in anatomical location (70) and may include more than one cortical area.

Considering all extrastriate visual cortical areas, our conclusion is that the current human pRF measures reveal fine-scale disparity responses and reflect binocular depth processing at different stages along the visual cortical pathway. Several previous studies have examined the effect of stimulus properties on retinotopic pRFs, such as the effect of using a colored stimulus (13), a moving stimulus (71), or a stimulus defined by differences of binocular depth (9). The present work successfully extends the pRF method to map tuning of RFs in nonretinotopic dimensions.

### Emergence of Sensitivity to Relative Disparity.

The great advantage of MR-imaging techniques is the opportunity to compare the response of multiple cortical areas simultaneously. Neurophysiology in macaques has established that there is a general tendency for the responses of higher-order cortical areas to be dominated by signals about relative depth (21) whereas the response of V1 neurons is completely governed by absolute disparity (50). Human population responses are consistent with this trend, although there are some complexities.

In practice, neuronal recording in macaques finds many cases of visual neurons that do not respond purely to relative disparity, even in higher visual areas. Often, neurons have response profiles with intermediate sensitivity that are a mixture of responsiveness to both absolute and relative disparity (52, 53). Thomas et al. (52) modeled the formation of relative disparity with a combinatorial model that is structurally similar to standard models for complex cells in the primary visual cortex and showed how such models could deliver intermediate sensitivity.

Even at the perceptual level, human vision does not deliver a pure relative depth signal from stereoscopic vision. Foulkes and Parker (64) introduced noise separately into absolute and relative disparities. There were careful controls to ensure that the effects of absolute disparity noise were not simply due to movement away from the fixation plane, which would remove the stimulus from the depth location with highest sensitivity to absolute depth. Foulkes (59) concluded that the perceptual signal is approximated with $w_{r}=0.8$ and $w_{a}=0.2$. A similar ratio of 4:1 in favor of relative disparity has been found recently in an extensive examination of this question across multiple spatial frequencies (58). On this basis, it would be predicted that even in the higher-order areas of the visual cortex, the balance is likely to be a weighting of 0.8 in favor of a response to relative disparity. This agrees with the outcome of modeling the human pRF responses in higher visual areas, such as LOC and V7.

A related issue is the so-called “absolute disparity anomaly” in human behavioral data, such that psychophysical judgments about absolute disparity are poor, even though it seems that relative disparity judgments are formed by combining the outputs of units sensitive to absolute disparity (72). This anomaly has also been found in earlier experiments in macaques that combined recording of V1 neurons with the performance of a psychophysical decision (35). In that case, V1 neurons outperformed the monkeys’ thresholds when the monkeys were required to make absolute disparity judgments. Both human and macaque studies therefore conclude that behavioral performance for judging absolute disparity is poorer than it should be. Two possible ways of addressing this paradox are: a) the primate visual system always takes a pessimistic view of the quality of vergence control, as poor vergence would disrupt absolute disparity but not relative disparity judgments or b) behavioral judgments about absolute disparity are not supported directly from V1 but may be supported from poorer-quality signals from other brain sites.

### Attentional Effects Unlikely to Generate pRFs.

Referring to Fig. 5, there are separated double peaks in the population distributions of pRF tuning curves, which are especially obvious in extrastriate cortical areas. It might be argued that these are driven wholly by the attention-grabbing properties of the stimulus. The segregation of the disk from the background, which occurs with suprathreshold nonzero disparities, is undoubtedly perceptually salient, which may possibly be enough to allocate extra attentional resources. The extra allocation of attention may be manifest as an enhancement of the BOLD response, as has been reported for other experimental paradigms.

The simple prediction based on perceptual salience driving attentional allocation is for a double-peaked pRF, each peak corresponding to a point of salient segregation of disk from the background. The double peaks in the population distributions are not produced by double-peaked responses at single vertices. Rather, they are produced by distinct sets of vertices, some exhibiting a peak at near disparities and others having a peak at far disparities. The analysis procedure for extraction of pRFs is incapable of producing a double-peaked tuning curve for a single vertex. The appearance of a double peak in the local HRF (Fig. 1*D*) is attributable to the double passage of the stimulus (forward and backward) within a single-peaked RF. Thus, if a peak in pRF tuning is driven by an allocation of extra attentional resources, then it must be an allocation that is depth specific.

Our experimental paradigm arranged for spatial attention to be continuously allocated to the disks, regardless of the depth plane in which they are situated. The observer is required to monitor the disks and report any slight changes in the contrast of the dots forming the disks, changes that occur unpredictably during the MR scan. There is therefore nothing to drive attention to any one depth plane. For feature attention to generate one of the prominent peaks at nonzero disparities, there would need to be a selective focus of attention toward either the near or the far depth plane. However, the perceptible changes in the depth of the disks that take place are always arranged symmetrically and simultaneously to near and far depth planes. There is therefore no driver for selective allocation of feature attention to near or far depth planes.

### Links to Neuronal Physiology.

The parallels we have found between the properties of MR-based pRFs and RFs recovered from neurophysiological recordings in macaques add to the evidence that the pRF procedures reveal interpretable measures of RF-like structures. In this respect, the extension to nonretinotopic stimulus dimensions is an important step forward. For more direct evidence that could link individual MR-based pRFs and recording from small groups of single neurons, a different approach would be needed. This would involve simultaneous measurement of the MR BOLD responses, while electrophysiological recording is in place.

A recent study has compared MR-based retinotopic pRFs with electrophysiological RFs in a group of 4 macaque monkeys, two of which were measured with MRI and the other two electrophysiologically using Utah array electrodes, with 14 arrays in V1 in one animal and two arrays in V4 in the other (10). The MR-based procedures produced a relationship between pRF size and pRF eccentricity that matched well the electrophysiological recordings of multiunit activity for both areas. For V1, these receptive fields mostly lay within the central 5 degrees of the visual field. It would be helpful to have comparisons directly within the same animals, including a greater range of eccentricities, as well as the exploration of nonretinotopic stimulus variables.

The responses of extrastriate neurons at large disparities appear to present a further puzzle, in that they respond to disparities larger than those represented in V1. These large disparity responses could conceivably be supported by monocular signals that pass out of V1 or elsewhere and become combined binocularly at downstream extrastriate sites (18, 27, 29). However, the requisite monocular signals are rarely observed. One attractive alternative is to suggest that the functional equivalent of an eye-specific response can be constructed, as needed, by local recombination of binocular signals arriving from V1. For example, one classic computational model converts left and right eye monocular signals in V1 to a pair of binocular signals L+R and L−R (73), which is a transformation that can clearly be reversed at higher levels to regenerate monocular responses. There is therefore no real difficulty in constructing receptive fields that represent large disparities in higher-order areas.

Several further issues intrude upon a direct comparison between BOLD-pRF measures and electrophysiology. The first is that the visual stimulus can never be optimized for all parameters of the visual sensitivity of a single cortical vertex during the mapping procedure. Classical electrophysiological recordings of single neurons are typically performed under conditions in which many other visual attributes of the stimulus have been optimized before a tuning curve for binocular depth is measured. These include basic geometric properties such as stimulus location, size, and spatial feature content, but also color, motion, texture, shading, and other visual content. Second, although we have working, computational models of how neural activations might be registered as changes in BOLD activity, present models are unlikely to reflect accurately the underlying physiological steps from visual excitation to hemodynamic vascular response. The third issue is potentially there may be uneven sampling of neural populations, which may arise from variations in the ability to detect BOLD responses (74) as well as from sampling bias from microelectrode recording. Fourth, some neurons identified from electrophysiology appear to have a tuning profile that is suppressive for a narrow range of binocular depths, often those centered on the fixation plane. These are the neurons identified by Poggio (24) as tuned inhibitory. Consequently, the BOLD signal for zero-disparity stimuli may be a mixture of excitation and suppression of neuronal firing, with the overall result that it may be weaker and less specific than the neurophysiology would predict. Implicit in the pRF results is the requirement for some clustering of binocular disparity responses in the visual cortex, which has been shown weakly for macaque V1 and strongly for V5 (27, 28). This is a question requiring further exploration.

## Conclusion

Our results advance the use of the pRF as a research tool for investigating binocular depth. The properties of pRFs that we have isolated from the human cortex show several strong similarities at the population level with electrophysiological recordings from the macaque visual cortex. This concordance further establishes the validity of the macaque as an important animal model of human binocular vision. Use of pRF modeling also provides more specific information about the preferred disparity and tuning of identifiable locations upon the neocortical surface, in comparison with earlier studies that used multivoxel pattern analysis to estimate the role of entire visual areas in depth perception (75, 76) or source imaging of visual evoked potentials (77).

Disorders of binocular vision remain one of the most persistent features of childhood and adolescent ophthalmology. Large programs of clinical care and monitoring are involved (78, 79) but a significant number of cases with amblyopia persist into adulthood with no favorable clinical outcome. Many of the individuals affected no longer receive clinical support in adulthood. Yet, there are several adverse comorbidities in this group, including a 100% increase in the risk of acquired total blindness due to loss of the better functioning eye (80) and, surprisingly also, increased risks of 25 to 30% for various cardio-metabolic disorders (81). The development of a reliable research tool for investigating binocular function using MR-methods that are validated with recordings from animal studies will hopefully provide insights and information that contribute to the reduction of these persistent cases.

## Materials and Methods

The MR-measurements were obtained from 10 healthy human participants with normal or corrected-to-normal vision (age range 19 to 45 y, mean age 31.4 y, 7 females). A detailed description of the scanning procedures is presented elsewhere (22).

Within the visual stimulus, the depth of the panel in each quadrant varied slowly over time following a periodic waveform that arranged for data acquisition to be concentrated on small depth differences close to the binocular fixation plane (20 log-scaled steps spanning −0.3° to +0.3° disparity from the fixation plane). As binocular depth in the quadrants changed, the cortical BOLD response tracked these changes. BOLD signals obtained under disparity stimulation were modeled with the matching disparity regressor using a one-dimensional pRF model.

### Neuronal Data from Electrophysiological Recordings.

For comparison with the pRF data analyzed here, we used data from earlier electrophysiological recordings of single neurons in the macaque visual cortex, area V1 (18, 28) and area V5/MT (20). These recordings were made from awake, behaving monkeys under regulated procedures granted by the UK Home Office (see original papers for full details).

### Consent.

The study received ethical approval from the University of Oxford Central University Research Ethics Committee (R53110/RE002) and informed written consent was obtained in accordance with the Declaration of Helsinki (1962) and its later amendments.

## Supplementary Material

Appendix 01 (PDF)

## Data Availability

Previously published data were used for this work. All other data are included in the manuscript and/or *SI Appendix*.
