# Supplementary material for: Receptive fields from single-neuron recording and MRI reveal similar information coding for binocular depth
Source: Proc Natl Acad Sci U S A. 2025 Nov 3;122(45):e2409893122. doi: 10.1073/pnas.2409893122 (PMC12625861; doi:10.1073/pnas.2409893122)
Supplement: Supplementary file 1 — Appendix 01 (PDF) [file pnas.2409893122.sapp.pdf]

Supplementary Material
for

Receptive fields from single neuron recording and magnetic resonance imaging reveal similar information coding for binocular depth.

Proceedings of the National Academy of Sciences

Andrew J. Parker[1,2,6], Ivan Alvarez[3,4], Alessandro Mancari[4,5], I. Betina Ip[3,4], Kristine Krug[1,2,6], Holly Bridge[3,4].

[1] Department of Sensory Physiology, Institute of Biology, Otto von Guericke University, Magdeburg, Germany
[2] Department Physiology, Anatomy and Genetics, University of Oxford, United Kingdom
[3] Oxford Centre for Functional MRI of the Brain (FMRIB), Centre for Integrative Neuroimaging, University of Oxford, Oxford, United Kingdom
[4] Nuffield Department of Clinical Neurosciences, University of Oxford, Oxford, United Kingdom
[5] Department of Translational Research on New Technologies in Medicine and Surgery, University of Pisa, Pisa, Italy
[6] Leibniz-Institute for Neurobiology, Magdeburg, Germany

Corresponding Author: Andrew J Parker **Email:** andrew.parker@ovgu.de
ORCID IDs
https://orcid.org/0000-0001-5800-0407 Andrew J Parker
https://orcid.org/0000-0003-2815-2666 Ivan Alvarez
https://orcid.org/0009-0002-7272-6049 Alessandro Mancari
https://orcid.org/0000-0003-3544-0711 I Betina Ip
https://orcid.org/0000-0001-7119-9350 Kristine Krug
https://orcid.org/0000-0002-8089-6198 Holly Bridge

S0. Extended materials and methods.

The MR-measurements were obtained from 10 healthy human participants with normal or corrected-to-normal vision (age range 19-45 years, mean age 31.4 years, 7 females). The participants had normal visual acuity (Snellen chart at 6 meters, <20/20 corrected) and stereoscopic vision (TNO test, correct detection at <60 arcsec; Frisby stereotest (http://frisbystereotest.co.uk/) correct detection at <40 arcsec). Each participant took part in up to three sessions: two MRI sessions for main data acquisition and one for retinotopic mapping. Three out of the ten participants already had retinotopic data available from a prior study and therefore only completed the two MRI sessions for main data acquisition. The study received ethical approval from the University of Oxford Central University Research Ethics Committee (R53110/RE002) and was conducted in accordance with the Declaration of Helsinki.

A detailed description of the scanning procedures is presented elsewhere (1). In brief, MR images were acquired on a 3T Prisma MRI system (Siemens Healthcare, Germany) using a 64-channel head coil (Siemens). Functional images were acquired with a gradient echo EPI sequence (TR = 1355 ms, TE = 32.4 ms, flip angle = 70°, 72 slices, resolution = 2 mm isotropic) with parallel multiband acceleration (MB factor = 4).

Within the visual stimulus, the depth of the panel in each quadrant varied slowly over time following a periodic waveform that arranged for data acquisition to be concentrated on small depth differences close to the binocular fixation plane (20 log-scaled steps spanning -0.3° to +0.3° disparity from the fixation plane). The time course of the waveforms was arranged in pairs across the four quadrants, so that when the depth was changing to points beyond the fixation plane for one pair, the depth of the other quadrants was changing to points nearer than fixation. The average binocular depth of the stimulus array was therefore always at the distance of the fixation plane (see Figure 1). Consequently, taken as a whole, the binocular stimulus provided a net zero drive for convergence or divergence of the eyes.

The binocular stimuli consisted of bright and dark dots, always balanced in number, presented on a grey background of intermediate luminance (see Figure 1C) and dynamically refreshed at a rate of 30Hz. Changes in the number or spatial distributions of dots changed the local contrast of the stimulus without changing the mean luminance of the display. For binocularly correlated stimuli, each bright or dark dot presented to the left eye was matched with a corresponding bright or dark dot to the right eye. This constraint applied for all values of binocular disparity, both zero and non-zero. For a binocularly anticorrelated stimulus, the spatial correspondence between dots was maintained but each bright dot in the left eye was assigned a corresponding dark dot in the right eye and each dark dot in the left eye was assigned a corresponding bright dot. Anti-correlation therefore specifies a geometric alignment of bright and dark dots between the two eyes and must be clearly distinguished from binocular uncorrelation, which specifies no systematic relationship of dot positions or brightness values between the two eyes.

To guard against long-term habituation from repetitive presentation of identical waveforms, the phase of the waveform for all four quadrants was flipped by π radian at unpredictable intervals. Participants were assigned a moderately demanding task requiring them to monitor and respond to changes in the contrast of the dots within each quadrant. This ensured a consistent allocation of attention to the visual stimulus during the changes in the binocular depth of the display. As binocular depth in the quadrants changed, the cortical BOLD response tracked these changes. BOLD signals obtained under disparity stimulation were modelled with the matching disparity regressor using a 1-dimensional pRF model.

Prior to this step, independent measurements of the HRF were obtained for each participant (2, 3), the cortical surface was approximated with a triangular mesh and a retinotopic pRF (*x* position*, y* position, location, and size) was measured for each vertex on the mesh. These retinotopic pRFs were used to assign boundaries between visual cortical areas and to identify the set of vertices whose *x,y* visual field co-ordinates fell within the quadrant stimulation of the binocular depth display.

Model predictions for a given quadrant aperture were generated by combining the disparity values shown during stimulation and a tuning curve for binocular depth, which was assumed to be a Gaussian defined by three parameters; peak disparity (μ), width of the tuning curve, expressed in standard deviations (σ) and an amplitude parameter (β). The model prediction was convolved with the participant-specific HRF and compared to the observed signal in an iterative optimization procedure (see Alvarez et al. (3)). Model performance was assessed with the coefficient of determination (R^2), as previously performed for measurement of 2-D spatial pRFs. Only vertices with model goodness-of-fit $R^2 > 0.1$ were retained for further analysis. Each retained vertex was characterized by the three fitted parameters of the Gaussian tuning curve for depth.

For about 15% of vertices with acceptable R^2 values, some estimates of parameters for the tuning curves emerged as clearly unrealistic. The following cases were eliminated from the data set: curves with an estimated value of peak disparity (μ) outside the range tested (-0.3 to 0.3 degrees); curves with an estimated tuning curve width (σ) narrower than the sampling interval for changes in the depth of the stereo stimuli (< 0.025 degree) or greater than the total range of disparities tested (>0.6 degree). The outcome of this data trimming is summarized in Table 1, which summarizes the numbers of recovered tuning curves for each cortical area: NVx is the total number of vertex points; NAVx is the total number of vertex points that show a valid pRF ($R^2 > 0.1$); NTrim is the number of vertex points after trimming unrealistic values of peak disparity (μ) and tuning curve width (σ). The statistical classification of pRFs was performed using the DBSCAN algorithm, available in MATLAB Statistics and Machine Learning Toolbox, R2024a.

Table S0.1 Number of active vertices by cortical area under binocularly correlated stimulation.

	hV1	hV2	hV3	hV4	hV5	hV3AB	hV7	hVOC	hLOC
NVx	35038	31601	23624	9139	13615	11623	7320	9406	19041
NAVx	10041	13406	12569	2908	7955	8877	5744	4021	14039
NTrim	8463	9895	8849	2131	6333	6754	3776	2978	9412

Neuronal data from electrophysiological recordings

For comparison with the pRF data analyzed here, we used data from earlier electrophysiological recordings of single neurons in macaque visual cortex, area V1 (4, 5) and area V5/MT (6). These recordings were made from awake, behaving monkeys under regulated procedures granted by the UK Home Office (see original papers for full details). From area V1, we used the plots of population sensitivity from Figs 12 & 13 in Prince, Cumming and Parker (5), as the calculations made there are equivalent to Fisher information under the assumption of negligible noise correlations. These calculations used the means and variances of the firing rates of single neurons from 180 recordings from V1 in two awake behaving macaque monkeys. The tuning curve data from single neurons were extracted after fitting the tuning curves with a Gabor function (the product of a Gaussian with a sinusoidal waveform). As in previous studies (7), it was found that the variance of the single neuron responses was roughly proportional to mean firing rate. This relationship

means that neuronal responses are more variable at higher firing rates. The standard approach is to apply a transform to the firing rate data to ensure that the variance is homogeneous (8). Therefore, the quantity used for calculation of neuronal discriminability was the square root of firing rate, which allows the responses of different neurons to be directly combined. Elsewhere in this paper, we build upon the finding that BOLD activations are more closely related to LFP and local synaptic activations, rather than to neuronal spiking activity (9). The human pRFs isolated here are fundamentally driven by BOLD responses of the cortex. Given the accelerating relationship between synaptic activation and neuronal firing delivered by exponents of 2 (10, 11), it is justifiable to compare human pRFs with the square root transform of electrophysiological tuning curves from macaque.

The electrophysiology data from macaque V5 are used to compare against the responses human pRFs from V5. This data set from two macaque monkeys was recorded from area V5 and consists of 140 isolated single neurons (6), which were pooled to compare the population responses to correlated and anticorrelated random dot patterns. As first observed by Julesz (12), the striking perceptual effect of binocular anti-correlation with random dot stimuli is that the appearance of stereoscopic depth is entirely lost. Neurons in the visual cortex often respond to anticorrelated stimuli with an inversion of the disparity tuning curve. Cumming and Parker (13) showed that the simple form of the binocular energy model (14) predicts this result but also predicts that the amplitudes of the response to correlation and anticorrelation should be the same, with only a change of sign. Experimentally, this is observed only rarely. The anticorrelated response is often substantially weaker. More realistic models insert non-linearities into combination of left and right eye responses, which reduce the amplitude of the response to anticorrelated stimuli (15). Qualitatively, any appearance of inversion of tuning functions with anticorrelation is a useful indicator that a receptive field can be modelled with some form of binocular energy model.

S1. Density-based clustering is associated with differences in the peak position of the disparity tuning curves of the pRFs

The density-based clustering analysis has the disadvantage of not providing direct confirmation that the discovered clusters have provable statistical significance. In this section, we examine in detail the clustering achieved for the data from V1 pooled across all nine participants for the binocularly correlated stimuli.

The pRF fitting procedure describes the disparity selectivity at each vertex with an estimate of the best-fitting Gaussian curve, resulting in four parameters for each vertex. Three of these parameters describe the Gaussian and the fourth is a performance measure (perf) for the quality of fit, which is the standard R-squared measure of variance explained. The three parameters for the Gaussian are the amplitude (beta), the location of the peak (mean) and the spread of the Gaussian(sigma). Only pRFs with a perf value greater than 0.1 are used for further analysis.

All four parameters were normalized prior to further analysis. These normalized parameters were employed in a one-way multivariate analysis of variance (MATLAB R2023b, `manova` function) to test which parameters are predictive of the classification provided by the density-based clustering. For the binocularly-correlated V1 data, the clustering algorithm provides two candidate clusters (here called, G1 and G2), leaving the other vertexes as not classified (NC). In total, there are 8463 valid pRFs from V1 across all 9 subjects. Of these, 4020 were assigned to G1, 3162 were assigned to G2 and the remaining 1281 were not classified NC. The outcome of the MANOVA is summarized in the Table below.

Table S1.1: one-way multivariate anova on V1 density-based classification

Source	DF	TestStatistic	Value	F	DFNum	DFDenom	pValue
NCG1G2	2	pillai	0.87529	1645.6	8	16916	0
		wilks	0.14213	3493.9	8	16914	0
		hotelling	5.9135	6250.9	8	16912	0
		roy	5.8927	12460	4	8458	0
Error	8460						
Total	8462						

```
1-way manova
Perf,Mean,Sigma,Beta ~ 1 + NCG1G2
```

There is evidence of a strong relationship between the classification from density-based clustering and the parameters delivered from the pRF modelling. The F-statistics are highly significant on a variety of possible tests. The next plots explore this relationship in more detail to reveal the main drivers of these differences. Figure S1 shows that the main driver of the relationship arises from the association between the mean value of the fitted Gaussians and the classification into G1 and G2. There is a weaker association between Sigma and the classification. These two confirm the visual impression on looking at the data plotted in the main paper. The classification is dominated by the position of the peak of the Gaussian. When this peak is also sharply defined (due to a smaller value of Sigma), then this further assists the separation by density-based clustering. Neither Beta nor Perf contribute substantially.

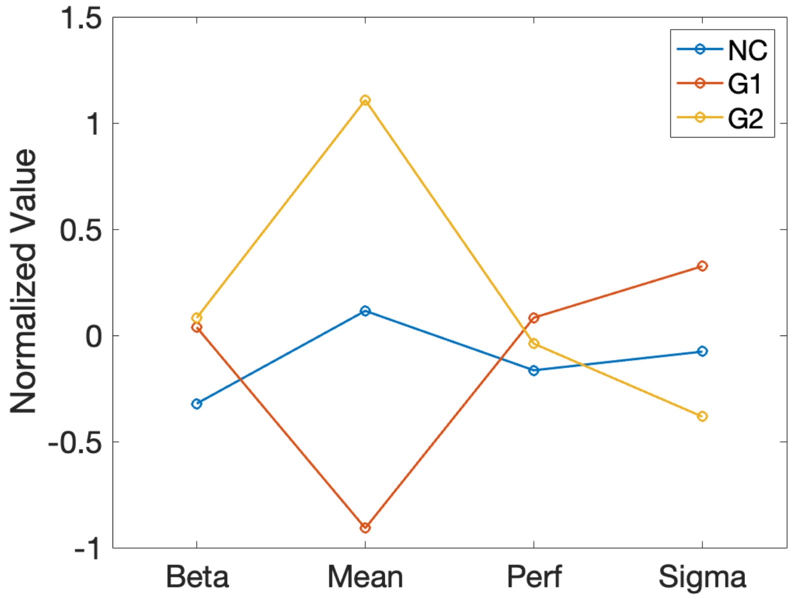

Figure S1.1 : Profile plot showing the associations between the 4 parameters of the pRF and the density-based classification. G1 and G2 are the two clusters from density-based clustering and NC is the residual unclassified data. The 'Mean' value from the pRF fits is the biggest driver of the association with a smaller contribution of 'Sigma'.

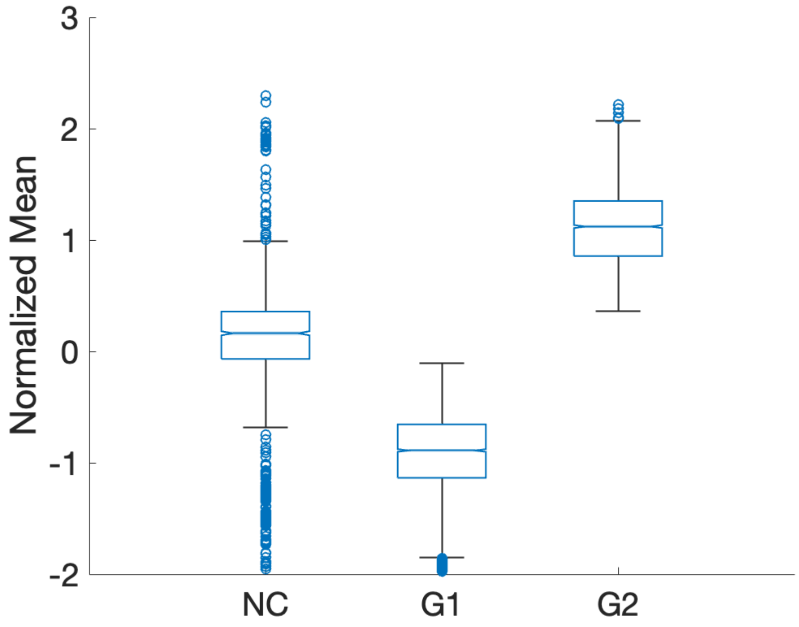

Figure S1.2: The value of the mean parameter from pRF modelling contributes substantially to separation into different clusters G1 and G2. NC is the unclassified data points.

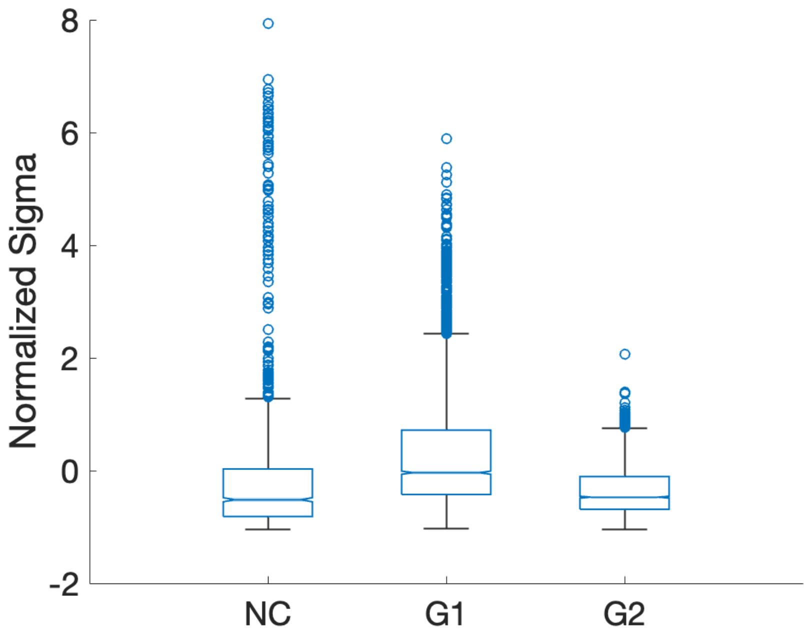

Figure S1.3: as Figure S1.2 but for sigma

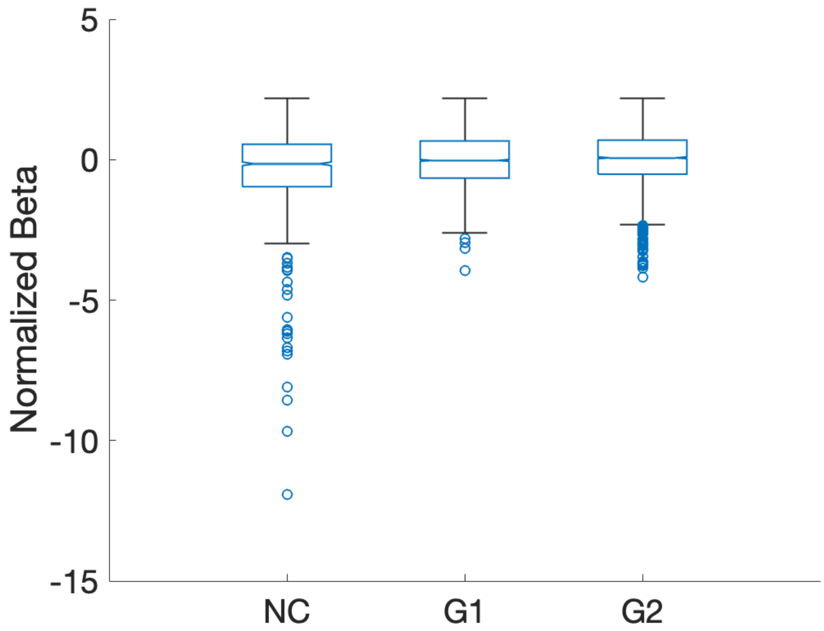

Figure S1.4: as Figures S1.2 and S1.3 but for Beta, amplitude of Gaussian

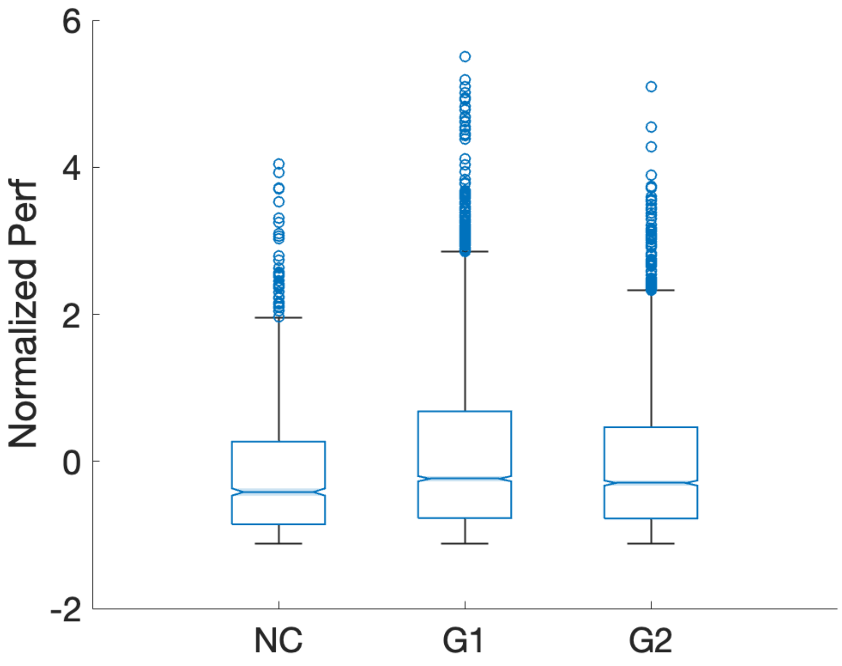

Figure S1.5: as Figures S1.2-S1.4, but for Perf, pRF model performance

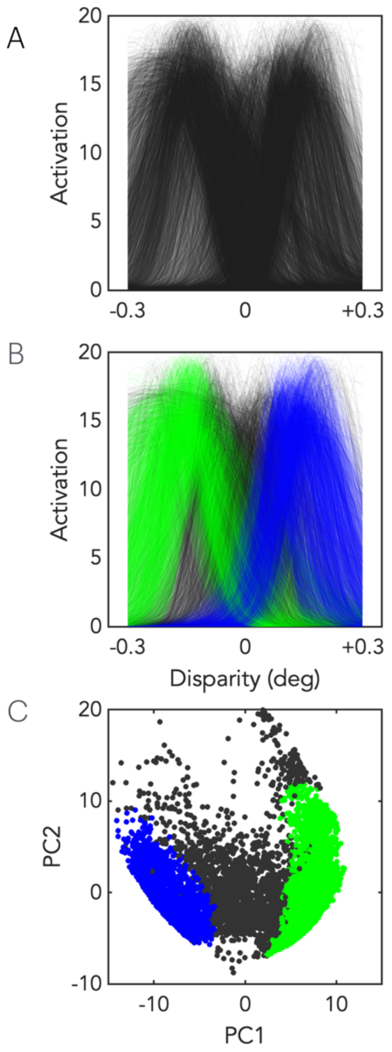

Figure S1.6: A: pRFs from hV1, each thin gray curve is the measured activation at a vertex on the meshed representation of the cortical surface. B: classification of the pRFs into different groups using density clustering. C: principal components analysis of the pRF distributions, color coded according to the density-based clustering analysis. The x and y axes show first and second principal components (PCA1 and PCA2).

S2: relative disparity in higher cortical areas emerges from tuning for absolute disparity in V1

In this section, we show that selectivity from relative disparity can emerge from the application of a simple combination rule to the responses of neurons that are individually sensitive to absolute disparity. This combination rule implements the energy model calculation at a higher order level to neurons that have already been made selective for absolute disparity by the binocular energy model applied to the inputs from left and right eyes. Westheimer (16) proposed that relative depth is computed by "a mechanism like a differential amplifier, whose output is a difference signal which has been decoupled from absolute values of the two inputs". We also show that there is a close equivalence between these two conceptualizations of how sensitivity to relative depth is computed. We consider the scenario in which the visual system is presented with two different absolute disparities d_1, d_2 , so that the relative disparity ψ is the difference between these two disparities, $\psi = d_1 - d_2$. We show that a simple difference on the weighted outputs of V1 is sufficient for computation of ψ, up to a proportional term.

The fundamental reason why a simple difference is similar to the energy model calculation arises from the structure of the energy model, originally developed for motion (17, 18) and adapted for binocular V1 neurons (14). Thomas, Cumming and Parker (19) further adapted this energy computation to model the circuitry that could lead to sensitivity to relative disparity. In this last version of the energy model, the two component inputs are pairs of neurons sensitive to absolute disparity with peak sensitivities at different disparities. In terms of the energy computation, these pairs correspond to the Gabor-filtered signal and its time-delayed version in the motion energy models and the inputs from left and right eyes in the binocular energy model.

In all cases, useful computation performed by the energy model is driven by the interaction term that arises when a pair of component responses are linearly added and then squared, thus

$$(L + R)^2 = L^2 + R^2 + 2LR$$

Equation 1

For a binocular neuron, the term $2LR$ models the interaction between the left L and right R inputs to the neuron, while the other terms relate only to each eye's input taken separately. Typically, the component responses L and R are computed twice over with the input passed through a pair of quadrature filters (as in Roe et al (20) Fig 2).

A V1 neuron tuned to absolute disparity d derives its sensitivity to disparity under the standard binocular energy model from this interaction term, which is calculated separately for each member of the quadrature pair: $L_0R_0 + L_{90}R_{90}$, where the subscripts refer to phase angle of the Gabor filters. All the other terms relate to the purely monocular responses. A full presentation of the binocular energy model for V1 neurons is found in Read et al (15). With Gabor filters for the left and right eye inputs, the energy model predicts a tuning curve for disparity with the form of a Gabor function.

In constructing a model that is sensitive to relative disparity, Thomas et al (19) set up a pair of model neurons A_b, A_f, described by a Gabor tuning curve, with each neuron maximally sensitive to different absolute disparities, d_f, d_b, where the subscripts f and b refer to foreground and background regions of the receptive field of neuron R. They found that greater and more specific sensitivity to relative disparity was achieved if each component neuron $\mathrm{A_f}, \mathrm{A_b}$ did not simply implement equation 1, which implies summation and squaring; rather the model subtracted out the monocular responses, $(L+R)^2 - L^2 - R^2$, which effectively isolates the interaction term. Thus

$$A = (L_0 + R_0)^2 + (L_{90} + R_{90})^2 - (L_0^2 + L_{90}^2) - (R_0^2 + R_{90}^2)$$

Equation 2

which reduces to

$$2(L_0 R_0 + L_{90} R_{90})$$

Equation 3

Applying the energy calculation to the outputs of A_f, A_b (thus $\left(A_f + A_b\right)^2$) gives the neuron R sensitivity to a range of relative disparities, with its peak response when $\psi = d_f - d_b$. There is a new higher level interaction term I_ψ, equivalent to equation (3). Subtracting out all terms other than this interaction term, we have $I_\psi = I_{\psi_0} + I_{\psi_{90}}$ where I_ψ is the sum of responses passed through the even and odd components of a quadrature pair of neurons tuned to absolute disparity. In the terminology of G Poggio (21), the even component derives from a V1 pair comprising a tuned-excitatory (TE) and a tuned inhibitory (TI) neuron and the odd component derives from a pair of near-far V1 neurons (NF).

The interaction term I has been studied intensively in earlier literature. It is found as equation 29 in Watson and Ahumada (17) and equation 5 in Fleet, Wagner and Heeger (22). For relative disparity, Thomas (23) showed that when the neuron R is stimulated by two disparities d_1, d_2 the equivalent terms can be written as

$$I_{\psi_0} = cos(d_1)\, cos(d_2)\, \phi(d_1, d_2, \sigma)$$

Equation 4

$$I_{\psi_{90}} = sin(d_1)\, sin(d_2)\, \phi(d_1, d_2, \sigma)$$

Equation 5

The combined sum of these two is

$$I_\psi = cos(d_1 - d_2)\, \phi(d_1, d_2, \sigma)$$

Equation 6

where $\phi(d_1, d_2, \sigma)$ is the product of two 1-D Gaussians, one a function of $d_1$and the other a function of d_2, with σ being the standard deviation of the Gaussian terms.

$I_\psi = 0$, when $d_1 = d_2$, but the important insight is that for small differences between $d_1$and d_2, $cos(\psi) \cong 1 - (\psi^2/2)$. In the code for the simulations, the response of a

relative disparity selective neuron is calculated as the unsigned value of the difference, $\|\psi\|$, between two neurons tuned for absolute disparity. We assume that the visual system is equipped with some neurons that are sensitive to $\mathrm{d}_1 - \mathrm{d}_2$ and others that are sensitive to $\mathrm{d}_2 - \mathrm{d}_1$, so we plot the unsigned difference of V1 outputs.

All three functions $\cos(\psi), 1 - (\psi^2/2)$ and $1 - \|\psi\|$ are even-symmetric around zero, so that, for small values of ψ, each function has a proportionate relationship with ψ. This means that the output of a model that takes the local difference between two neurons that respond to absolute disparity varies its output proportionately according to the size of ψ, the relative disparity. In this respect, the energy model for relative disparity (19) and the differencing model (16) are closely similar.

In predicting the response of higher order cortical areas, we use a blended model of mixed responsiveness to absolute and relative disparity, $w_a * A + w_r * R$, where A is the original binocular energy model for V1 neurons and R is the higher order energy model proposed by Thomas et al (19). We take V1 activity as completely dominated by responses to absolute disparity A. Predicted responsiveness to relative disparity R is taken from the unsigned difference of V1 outputs. We find that different values of the weights $\mathrm{w_a}$, $\mathrm{w_r}$ are needed to match the response profile of different visual cortical areas, with a greater weight for relative disparity in higher order areas.

Like the binocular energy model for V1 neurons, the higher order energy model predicts that the tuning for relative disparity should be narrower than the tuning for absolute disparity. The reason is that the product term introduced above $\phi(d_1, d_2, \sigma)$ is narrower than the component Gaussians. Thus, if each component Gaussian is taken as identical, $\exp[-(x^2/\sigma^2)]$, the product pair is $\exp[-(2x^2/\sigma^2)]$, leading to the prediction is that the tuning for relative disparity should be narrower by a factor of $1/\sqrt{2}$. This prediction is discussed in the main paper and illustrated in Figure S2.1, where it is shown that the degree of narrowing is progressive from V1, through V2 to higher order areas. While this outcome is consistent with a greater weight for relative disparity in higher order areas, the observed degree of narrowing is greater than the theoretical factor of $1/\sqrt{2}$. This discrepancy cannot be explained by differential weighting of relative and absolute disparity signals. Narrowing by greater than $1/\sqrt{2}$ could be due to multiple hierarchical stages, each stage performing a higher order energy computation. Alternatively, the explanation may lie with inhibitory signals that enhance the contrast between RFs tuned to different ranges of disparity.

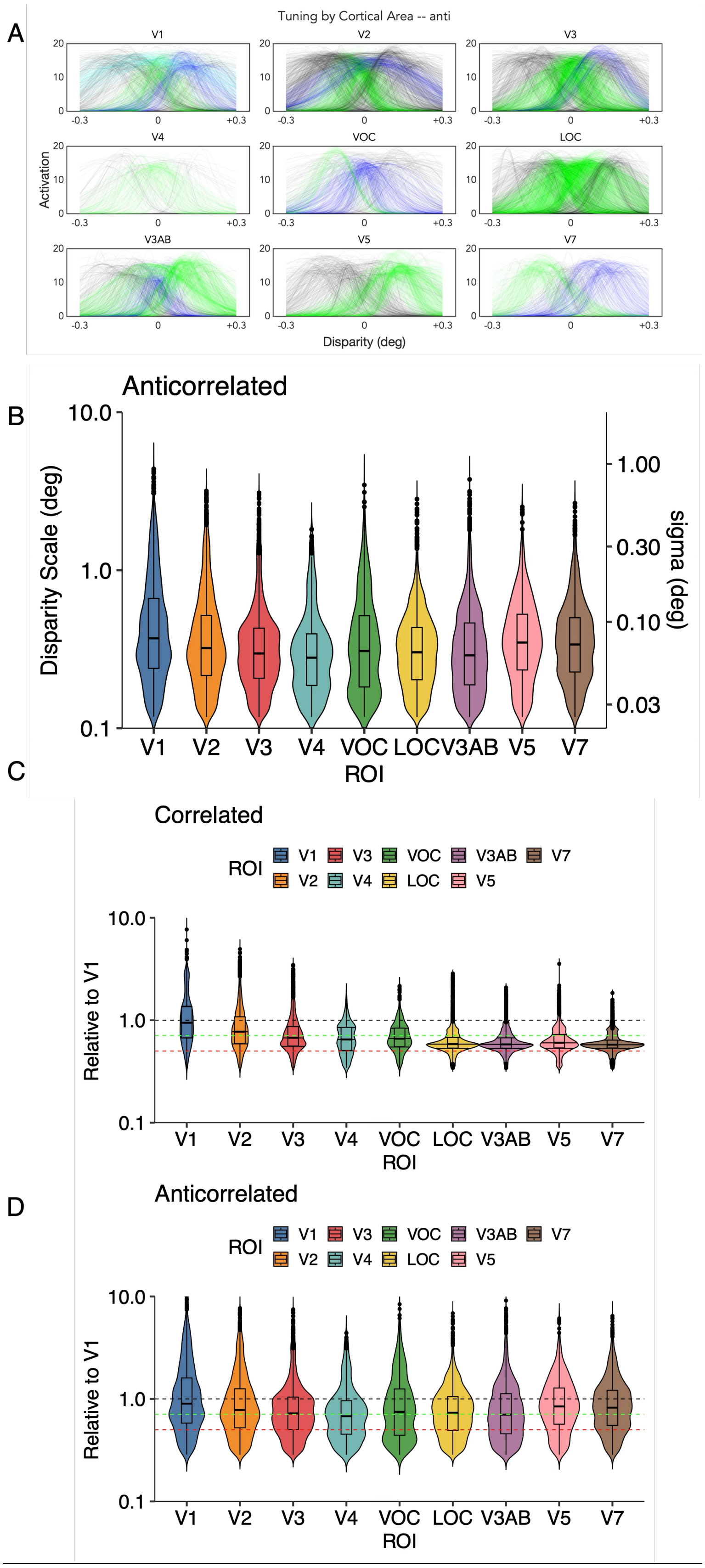

Figure S2.1 A,B: equivalent of Figures 3A and 3B from main paper but for anti-correlated stimulation. C,D: violin plots of disparity scale by cortical area for correlated (C) and anti-correlated (D) stimuli, after normalization by mean value for human V1. Dashed lines show unity (Black), narrowing by factor of $1/\sqrt{2}$ (Green: see section S2 for model prediction) and narrowing by 0.5 (Red).

S3: Vertex-by-vertex comparisons of peak response to correlated and anti-correlated disparities and of correlated responses with quality of fit.

Figure S3.1 expands on the relationship shown in Figure 4 (main paper) for pRFs for cortical area hV5/MT. Here the outcome for the full set of areas presented. The results for some cortical areas appear to show a group of pRFs that always have response to anti-correlated disparities at near zero disparity but have a widespread set of preferred disparities for correlated disparities. This relationship needs further testing but our current working hypothesis would be that this grouping may be related to the electrophysiological classes of tuned-inhibitory neurons identified by G. Poggio (21).

Figure S3.2 shows there is no evidence of a bias towards default values from the pRF fitting procedure as data quality (CRsq) decreases.

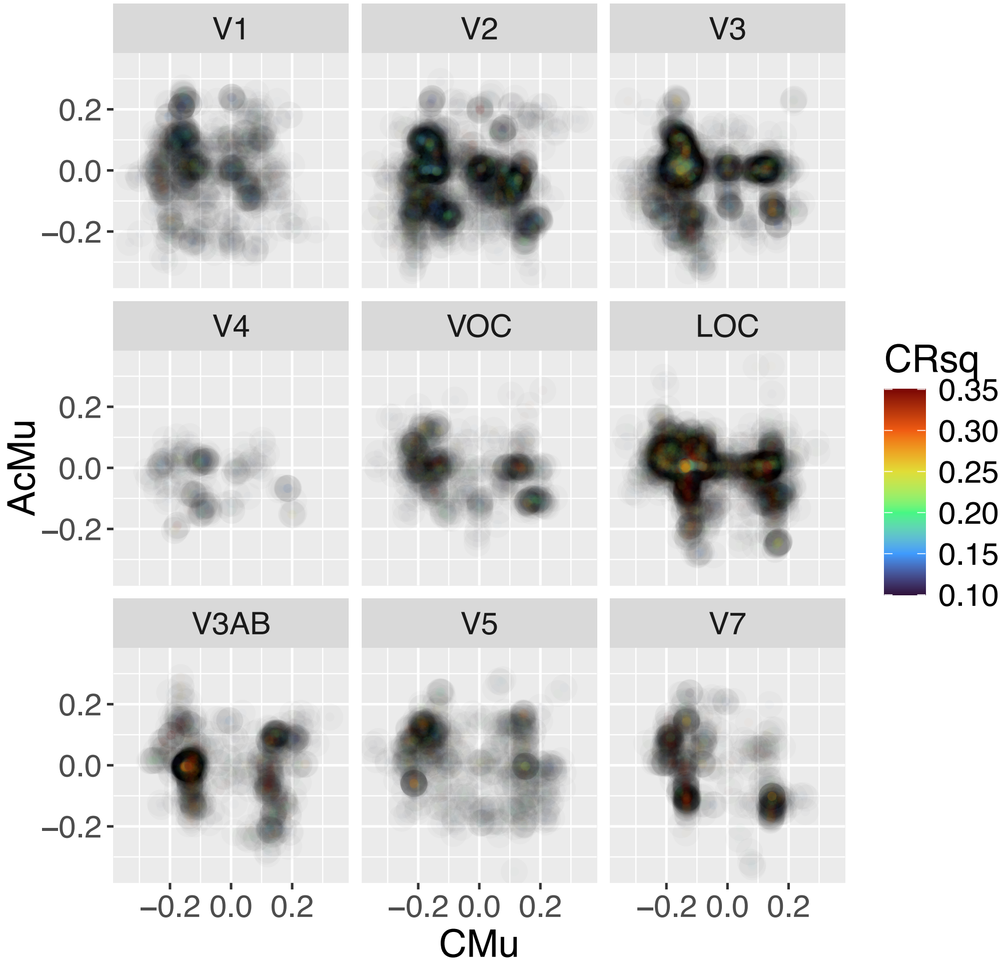

Figure S3.1 Plot of preferred disparity for human pRFs for correlated (CMu) and anticorrelated (AcMu), making a vertex-by-vertex comparison for all vertices for which $R^2 > 0.1$ for both correlated and anti-correlated response. X and Y axis units are in degrees of disparity.

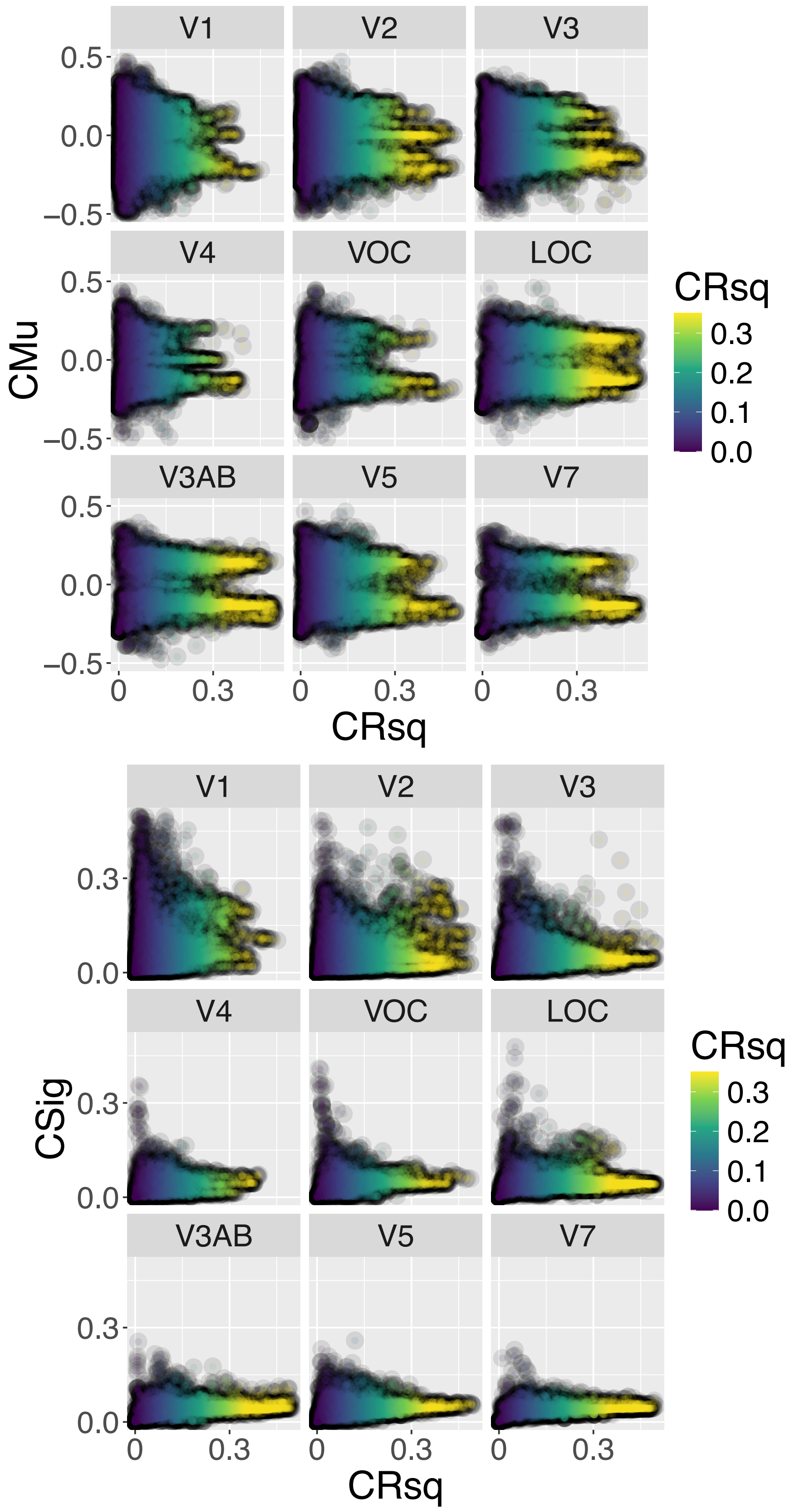

Figure S3.2 Relationship of mean (CMu) and sigma (CSig) for fitted Gaussians with quality of fit (CRsq) for all vertices in response to correlated disparity with no thresholding of CRsq.

References

1. I. Alvarez, A. Mancari, I. B. Ip, A. J. Parker, H. Bridge, Characterising human disparity tuning properties using population receptive field mapping. *The Journal of Neuroscience* 10.1523/JNEUROSCI.0795-24.2025, e0795242025 (2025).
2. I. Alvarez, S. A. Hurley, A. J. Parker, H. Bridge, Human primary visual cortex shows larger population receptive fields for binocular disparity-defined stimuli. *Brain Struct Funct* 10.1007/s00429-021-02351-3 (2021).
3. I. Alvarez, B. De Haas, C. Clark, G. Rees, D. Schwarzkopf, Comparing different stimulus configurations for population receptive field mapping in human fMRI. *Front Hum Neurosci* **9** (2015).
4. S. J. D. Prince, A. D. Pointon, B. G. Cumming, A. J. Parker, Quantitative analysis of the responses of V1 neurons to horizontal disparity in dynamic random-dot stereograms. *Journal of neurophysiology* **87**, 191-208 (2002).
5. S. J. D. Prince, B. G. Cumming, A. J. Parker, Range and mechanism of encoding of horizontal disparity in macaque V1. *Journal of neurophysiology* **87**, 209-221 (2002).
6. K. Krug, B. G. Cumming, A. J. Parker, Comparing perceptual signals of single V5/MT neurons in two binocular depth tasks. *Journal of neurophysiology* **92**, 1586-1596 (2004).
7. D. J. Tolhurst, J. A. Movshon, I. D. Thompson, The Dependence of Response Amplitude and Variance of Cat Visual Cortical-Neurons on Stimulus Contrast. *Experimental Brain Research* **41**, 414-419 (1981).
8. P. Armitage, G. Berry, *Statistical Methods in Medical Research* (John Wiley, New York, ed. 3rd edition, 1994).
9. N. K. Logothetis, J. Pauls, M. Augath, T. Trinath, A. Oeltermann, Neurophysiological investigation of the basis of the fMRI signal. *Nature* **412**, 150-157 (2001).
10. A. Anzai, I. Ohzawa, R. D. Freeman, Neural mechanisms for processing binocular information I. Simple cells. *Journal of neurophysiology* **82**, 891-908 (1999).
11. D. J. Heeger, Modeling Simple-Cell Direction Selectivity with Normalized, Half-Squared, Linear-Operators. *Journal of neurophysiology* **70**, 1885-1898 (1993).
12. B. Julesz, Binocular Depth Perception of Computer-Generated Patterns. *Bell System Technical Journal* **39**, 1125-1162 (1960).
13. B. G. Cumming, A. J. Parker, Responses of primary visual cortical neurons to binocular disparity without depth perception. *Nature* **389**, 280-283 (1997).
14. I. Ohzawa, G. C. DeAngelis, R. D. Freeman, Stereoscopic Depth Discrimination in the Visual Cortex: Neurons Ideally Suited as Disparity Detectors. *Science* **249**, 1037-1041 (1990).
15. J. C. A. Read, A. J. Parker, B. G. Cumming, A simple model accounts for the response of disparity-tuned V1 neurons to anticorrelated images. *Visual Neuroscience* **19**, 735-753 (2002).
16. G. Westheimer, Cooperative Neural Processes Involved in Stereoscopic Acuity. *Experimental Brain Research* **36**, 585-597 (1979).

17. A. B. Watson, A. J. Ahumada, Model of human visual-motion sensing. *J. Opt. Soc. Am. A* **2**, 322-342 (1985).
18. E. H. Adelson, J. R. Bergen, Spatiotemporal energy models for the perception of motion. *J. Opt. Soc. Am. A* **2**, 284-299 (1985).
19. O. M. Thomas, B. G. Cumming, A. J. Parker, A specialization for relative disparity in V2. *Nature Neuroscience* **5**, 472-478 (2002).
20. A. W. Roe, A. J. Parker, R. T. Born, G. C. DeAngelis, Disparity Channels in Early Vision. *J. Neurosci.* **27**, 11820-11831 (2007).
21. G. F. Poggio, Mechanisms of Stereopsis in Monkey Visual-Cortex. *Cerebral Cortex* **5**, 193-204 (1995).
22. D. J. Fleet, H. Wagner, D. J. Heeger, Neural encoding of binocular disparity: Energy models, position shifts and phase shifts. *Vision research* **36**, 1839-1857 (1996).
23. O. M. Thomas (2001) Neuronal responses to absolute and relative disparities in cortical area V2 (Doctoral Thesis). in *University Laboratory of Physiology* (University of Oxford), p 156.
